# How to recruit teachers for hard-to-staff schools: A systematic review of evidence from low- and middle-income countries

**DOI:** 10.1016/j.econedurev.2023.102430

**Published:** 2023-08

**Authors:** David K. Evans, Amina Mendez Acosta

**Affiliations:** Center for Global Development, USA

**Keywords:** Education, Teachers, Hard-to-staff schools, Low- and middle-income countries

## Abstract

Education systems struggle to staff schools in rural areas or in areas with high concentrations of poverty. Potential policy solutions include financial incentives, mandatory rotations, and local recruitment drives, among others. First, this systematic review provides evidence on challenges with teacher staffing in certain types of schools. We observe lower teacher skill and higher teacher absence in rural areas in many countries. Second, the review synthesizes available experimental and quasi-experimental studies of government-implemented policies to increase the quantity or quality of teachers in hard-to-staff schools in low- or middle-income countries. Financial incentives—the most evaluated policies—are often effective at increasing the supply or reducing the turnover of teachers in hard-to-staff schools, and well-designed incentives can also increase the quality of teachers in these schools. Impacts on student outcomes are often positive. Although there are fewer evaluations, behavioral and informational interventions have been cost-effective in reducing vacancies in two countries.

## 1 Introduction

Schools in rural areas and schools in areas with high concentrations of poverty or poor performance are difficult to staff. In low- and middle-income countries in particular, rural schools and the communities that house them may lack an array of amenities, such as quality housing and electricity. In urban areas, schools in high-poverty neighborhoods likewise may have less discretionary funding or teaching aids. These factors and others make recruiting and then retaining teachers—especially high-quality teachers—a consistent challenge. Dozens of low- and middle-income countries have implemented policies to increase the supply of teachers in hard-to-staff schools, including financial incentives, additional training, a faster track towards promotion, mandatory rotations, and subsidized housing (Elacqua et al., 2018; McEwan, 1999; Pugatch & Schroeder, 2014). More recently, governments have experimented with behavioral nudges to encourage teachers to select hard-to-staff schools (Ajzenman et al., 2021a; Ajzenman et al., 2021b). How effective are these strategies?

This review examines the available empirical evidence measuring the effectiveness of government-implemented policies to recruit and retain teachers to rural or otherwise hard-to-staff schools in low- and middle-income countries. To start, we summarize the cross-country challenge of recruiting teachers to difficult posts and present new evidence on teacher quality in hard-to-staff areas. We then outline the policy options available to address this challenge. Next, we present a systematic search that identified over 8,000 potential studies, of which 15 were experimental or quasi-experimental evaluations of policies to boost teacher recruitment or retention in schools. All of those studies were of interventions in Africa, Latin America, or South Asia. Twelve were of financial incentives to teachers and three were of other policies.

We next examine impacts of these policies on teachers and on students, with a separate analysis of results on the proportion of teachers who are women. Together with the results, we summarize the evidence on costs of these programs and findings from non-experimental studies with weaker evidence of causal effects, including surveys of teachers, which provide insight into stated preferences and experiences in a broader range of countries. Finally, we discuss common implementation challenges, methodological considerations, limitations to this work, and how our findings relate to those in high-income countries.

We find that most financial incentive programs have positive impacts on teacher outcomes, ranging from increasing placement in hard-to-staff schools to reducing turnover. Not all programs were successful: for example, one particularly small bonus in Brazil had no impact. Effects were sometimes concentrated in particular sub-groups: an incentive program in Peru improved recruitment, but only for short-term positions. One intervention—in Chile—focused explicitly on increasing the quality of teachers in hard-to-staff schools: that program dramatically increased retention for teachers already working in the disadvantaged schools at baseline, but not recruitment to those schools. Two other programs not explicitly designed to increase quality (in Peru and the Gambia) also boosted measures of teacher quality. There is evidence of implementation challenges in many of the programs, and evidence from Peru suggests that even when financial incentives are effective at boosting recruitment or retention, they are unlikely to close the existing gaps in teacher supply and quality at plausible spending levels.

Most evaluations of non-financial programs show positive impacts on teacher outcomes. Both informational and behavioral interventions were effective: one, in Chile, provided information either about an existing financial incentive program or emphasizing the good that teachers do, and another, in Ecuador, simply put hard-to-staff schools at the top of the list of schools to which teachers could choose to apply. Both interventions increased placements in hard-to-staff schools at low (or almost zero) cost. These are cost-effective complements to other efforts to staff rural or high-poverty schools. The final study, a recruitment drive for additional teachers in short-staffed schools in India—in which the federal government financed the teacher contracts for the first few years—did not increase teacher recruitment, but it did redistribute teachers from larger schools to smaller schools.

We find at least some positive impacts on student learning in most studies that report that outcome. (Only 9 of the 15 studies report student outcomes.) Some of the impacts are driven by sub-groups: an incentive program in Brazil improved student outcomes only for initially low performers, a program in Zambia significantly improved test scores for boys but not for girls, and an incentive program in Gambia improved student outcomes consistently only for better-off students.

Another key finding of the study is just how little evidence exists on the effectiveness of policies besides financial incentives to recruit or retain teachers in hard-to-staff schools, despite the fact that countries implement a wide array of policies and that teachers signal support for such alternative policies.

This review complements earlier work on recruiting and retaining teachers in hard-to-staff areas. A recent review of evidence on teachers that is almost entirely focused on high-income countries (See, Morris, Gorard, & El Soufi, 2020) also finds that financial incentives can be effective in recruiting teachers in disadvantaged schools. This paper also complements existing synthesis work on hard-to-staff schools in low- and middle-income countries: Elacqua et al. (2018) document a wide array of policies to attract teachers to vulnerable schools across Latin America, and Bertoni et al. (2018) discuss policies in Chile, Colombia, and Peru in greater detail. Related research seeks to document efforts to facilitate more even distribution of teachers across schools, without a focus on hard-to-staff schools per se (Bashir et al., 2018; Datta & Kingdon, 2021; Majgaard & Mingat, 2012).^1^

Finally, this paper contributes to the literature on teacher labor markets in low- and middle-income countries more broadly (Crawfurd & Pugatch, 2022), especially teacher compensation (Evans, Yuan & Filmer, 2022; Mizala & Ñopo, 2016). Indeed, several studies have used discontinuities for hardship pay to identify the impact of unconditional salary increases for teachers (Chelwa, Pellicer & Maboshe, 2019; Pugatch & Schroeder, 2018; Chacón & Peña, 2017), complementing other work on teacher salary increases (De Ree et al., 2018). A richer understanding of how to staff schools with challenging working conditions contributes to a fuller understanding of how to build an effective teacher workforce to deliver education well.

## 2 The problem of attracting and retaining sufficient teachers in hard-to-staff schools

Hard-to-staff schools face several important staffing problems. First, they face shortages of teachers, which may arise from either a failure of candidates to apply for these vacancies, a higher rate of turnover among teachers at those schools, or both. Second, they face a shortage of high-quality teaching candidates. Even if systems manage to staff rural schools or high-poverty schools, they may principally have to staff them with novice teachers, less motivated teachers, less skilled teachers, or contract teachers that do not yet meet the minimum official standards to be permanent teachers. Third, they may face shortages of teachers in other desirable categories, such as women teachers or teachers in key subjects (such as math and science at the secondary level).

Conceptually, this is because hard-to-staff schools tend to lack amenities that urban or low-poverty schools tend to have, thus reducing the desirability of working in a hard-to-staff school (McEwan, 1999). Rural schools are, by their nature, further from the comforts of urban life. Teaching in high-poverty schools, although they may be located in urban areas, may include increased exposure to crime on the way to and from school. Teachers in schools that are further from district or provincial centers may have more difficulty in accessing teacher professional development programs provided by the government or additional degrees and trainings available in universities. Rural and otherwise high-poverty schools also often have fewer complements for teachers to use in their work. Our analysis of differences between urban and rural schools—with rural schools being one common category of hard-to-staff schools—in eight African countries shows that schools in rural areas generally have fewer educational inputs.^2^ For example, in no country in that sample are students in rural schools more likely to have an exercise book than students in urban schools (Fig. A.1). Similarly, there are more countries in which rural students are significantly less likely to have a functioning blackboard than urban students than the reverse (Fig. A.2). Limited teaching resources may also contribute to lower student outcomes such as test scores, which in turn could limit teacher promotion in contexts where teacher advancement is based on measures of student learning. These challenges make teaching in hard-to-staff schools less desirable, and this is born out by the evidence we find across countries.

While challenges in staffing hard-to-staff schools are almost universal, the degree and precise nature of the challenges will naturally depend on the country context: for example, in Kenya, about two-thirds of schools are in rural areas while that number rises to nine out of ten schools in Madagascar. We discuss the variation in rurality in more detail in Appendix Section A.2.

### 2.1 Quantity of teachers

Many studies demonstrate the magnitude of the problem of recruiting and retaining teachers in hard-to-staff schools (Table 1 Panel A).^3^ In Peru, teacher candidates who score above a required threshold on a knowledge test may list all the schools they would be interested in teaching at as part of the teacher-school matching process. Schools in the most rural areas, with high levels of poverty, further from the provincial capital, or with lower student test scores were all more likely to have zero candidates select them (Ajzenman et al., 2021a). In a 2018 teacher survey, also in Peru, only 6.5 percent of urban teachers indicated a willingness to relocate to rural areas without additional compensation, and almost a quarter of teachers indicated they would not relocate under any circumstances (Castro & Esposito, 2022). In Brazil, schools with a lower socioeconomic index and poorer students have higher teacher turn-over rates, fewer teachers on permanent contracts, and generally worse teacher shortages (Camelo & Ponczek, 2021; Rosa, 2019). In India (albeit in a characterization that is now dated), almost 30 percent of India's half million primary schools were one-teacher schools in 1987 (Chin, 2005).

**Table 1:** Characterization of the Problem.

*Panel A: Quantity of teachers*		
Ajzenman et al. (2021a)	Peru	“Out of the 12,300 public schools that had vacancies in the 24 regions of Peru in 2019, 6424 (52%) were not selected by any candidate at the national stage. The difference in terms of observable characteristics between these two groups of schools is striking and illustrates teacher preferences for more advantaged institutions: those not selected are notably more rural, farther from the province capital, with less access to basic services, and with a greater proportion of low-performing students.”
Ajzenman et al. (2021b)	Ecuador	“Ecuador's teacher selection process still generates some inefficiencies and inequities. While some schools receive more applications than available vacancies, others struggle to attract applicants. As a result, a large proportion of teaching positions remain unfilled at the end of the process, and a number of candidates are unable to secure a job offer.”
Cabrera and Webbink (2020)	Uruguay	“In 2005, at the start of the program… schools in areas with a lower poverty score have a more experienced teaching staff.”
Camelo and Poczek (2021)	Brazil	Schools with low socioeconomic index “had higher turnover rates before the program (almost 53%)…had more students with worse profiles: lower average scores on proficiency exams, a higher proportion of poor performers, and less-educated parents… [and] had less-experienced teachers and fewer permanent contract ones.”
Castro and Esposito (2022)	Peru	“The 2018 National Teacher Survey showed that only 6.5% of teachers working in public schools in urban areas would be willing to accept a position in a rural school without any additional compensation, and 24.1% would not be willing to accept it under any circumstance. As a result, rural schools face difficulties attracting and retaining teachers. In fact, teachers in urban areas accumulate, on average, 50% more years of experience working in the same school than teachers located in the rural areas.”
Chelwa et al. (2019)	Zambia	“According to the Ministry of Education, in any given year 7% of the teaching staff in rural areas leave versus 3% in urban areas. Similarly, the tenure of teachers in rural schools is on average 2 years shorter than it is in urban schools.” Pre-treatment data from the sample confirms this trend: rural areas have rate of teachers transferring out (7.8% vs. 5.4% in urban areas) and lower average teacher tenure (10.0 years vs 11.2 years in urban areas).
Chin (2005))	India	At the time of program implementation, almost 30% of India's primary half million primary schools were one-teacher schools.
Pugatch and Schroeder (2014; 2018)	Gambia	“Female teachers are dramatically under-represented in hardship schools…with a 10 percentage points lower share of the overall teaching corps and a qualified female teacher-pupil ratio that is 50% lower.”
Rosa (2019)	Brazil	In the 2010 teacher recruitment cycle in São Paulo, Brazil, schools requested to fill 6336 positions but only 4205 candidates passed the exam and only 3643 are matched to schools. “Teacher shortage is larger in areas where the socioeconomic status of students is lower.”
Swai (2013)	Tanzania	“Since 1961, the Rukwa and Kigoma regions consistently face teacher shortages and high teacher demands, a situation created by limited mechanisms for motivating teachers to accept teaching positions and stay working in the region.”
*Panel B: Quality of teachers*		
Bobba et al. (2022)	Peru	“Teachers at rural schools are 20% less likely to pass the requirements set by the government for permanent teachers (competent teachers) and are twice as likely to lack teaching credentials (non-certified teachers).”
Elacqua et al. (2022)	Chile	More talented teachers as measured by test scores and teacher portfolio “are less likely to work in rural schools, municipal schools, and disadvantaged schools… [and] are less likely to leave the system in two years and less likely to transfer to another school.”
Hinze-Pifer and Méndez (2016)	Chile	The authors cite existing research that shows that private schools attract better skilled teachers (Behrman et al. 2016) and that teachers who studied at less selective universities tend to teach in disadvantaged schools (Ortúzar et al., 2009).
Urquiola and Vegas (2005)	Bolivia	“16 percent of urban teachers lack formal training, whereas in the provincial and rural areas, 24 and 29 percent, respectively, are in a similar situation”

### 2.2 Quality of teachers and teacher performance

Hard-to-staff schools face an additional challenge beyond simply unfilled vacancies. When they do fill their vacancies, they often have more poorly qualified teachers (Table 1 Panel B). In countries with centralized teacher labor markets, this may be because more qualified teachers sometimes have more choice over where they are placed. In countries with localized teacher labor markets, more qualified teachers may have more job offers.

For example, in Chile, teachers who won the pedagogical excellence award (based on teacher test scores and teacher portfolio) were less likely to work in rural or disadvantaged schools (Elacqua et al., 2022). Also in Chile, teachers who were trained at more selective universities were less likely to teach in disadvantaged schools (Ortúzar et al., 2009). In Peru, rural teachers were 20 percent less likely to fulfill eligibility requirements for permanent positions and were twice as likely to lack required formal teaching credentials (Bobba et al., 2022). In Bolivia, 16 percent of urban teachers did not have formal training, compared to 24 percent in provincial areas and 29 percent in rural areas (Urquiola & Vegas, 2005). In a descriptive study from Ghana, Gad (2015) reports that in the country's Northern region, “about 404 schools do not have a single trained teacher, leaving the running of the educational establishments in the care of volunteers and untrained teaching personnel.”

**Fig. 1:**
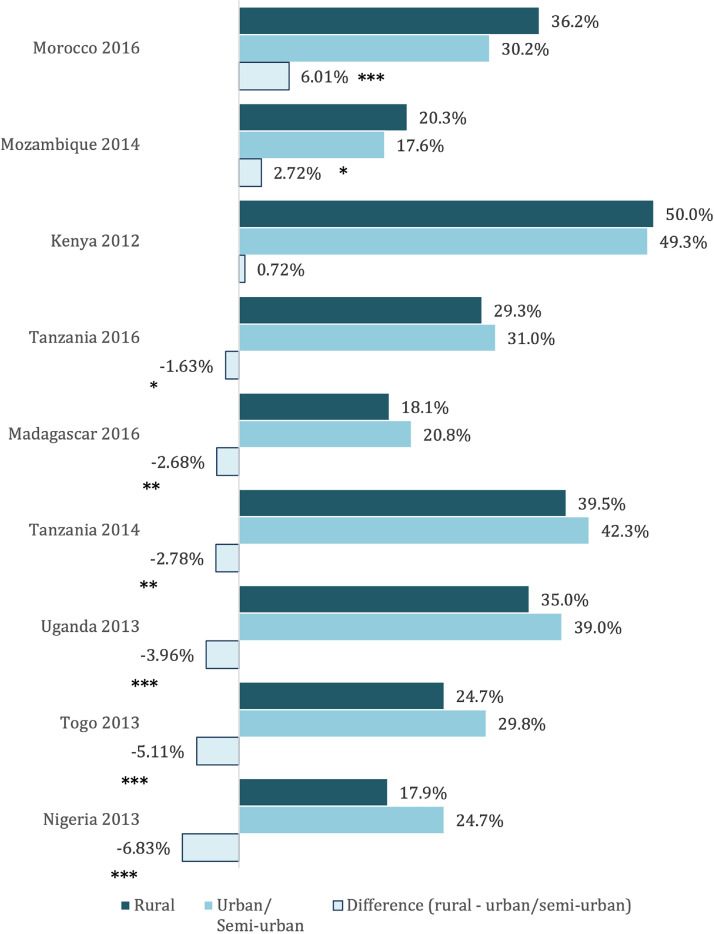
Teacher Competency Score in Urban and Semi-Urban vs. Rural Areas in Selected Low- and Middle-Income Countries.

*Source*: Authors’ analysis using data from the World Bank’s Service Delivery Indicators Education Survey.*Note*: The teacher competency score is the percent answered correctly in task-based assessments of teachers’ literacy, numeracy and pedagogy skills based on lower-primary level curriculum (scores are out of 100 percent). Averages are first computed at the school-level and aggregated at the country level using school-level weights. Differences in rural and urban/semi-urban rates are tested using bivariate regressions with school-level weights and robust standard errors and are presented here with statistical significance: * significant at 10%; ** significant at 5%; *** significant at 1%.

Our analysis of differences between urban and rural schools in eight African countries bears out a similar pattern, albeit not universally. For the countries in this sample, teacher competence is a challenge regardless of school location (Bold et al., 2017), but in five out of eight countries (Madagascar, Nigeria, Tanzania, Togo, and Uganda), teachers in rural areas score lower on tests of math, language, and pedagogy (Fig. 1). Appendix Tables A.1–A.3 show a similar pattern for each test (language, numeracy, and pedagogy), with rural teachers most consistently scoring lower in language. Differences in most countries are modest: only two are larger than five percentage points, and none are larger than ten percentage points.

**Fig. 2:**
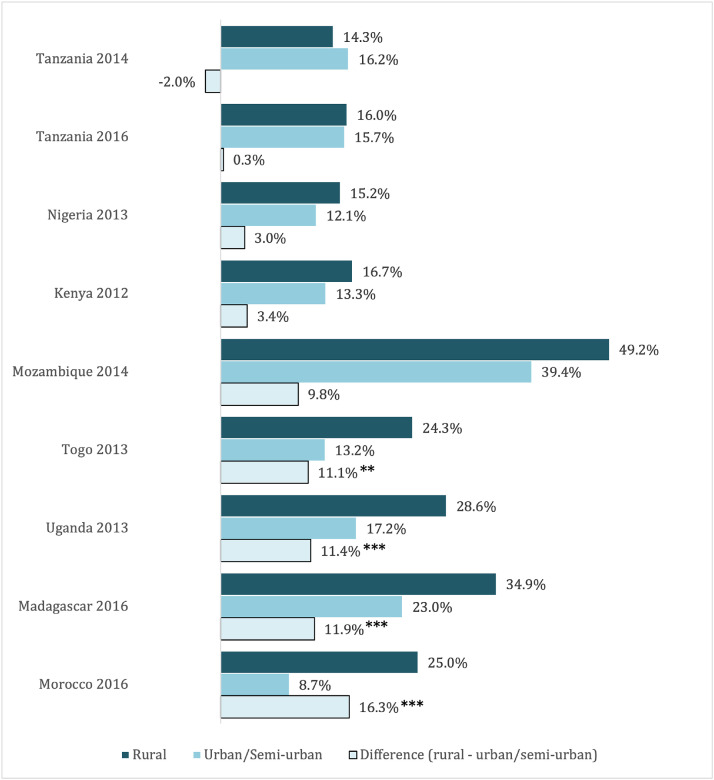
Teacher Absenteeism Rates in Urban and Semi-Urban vs. Rural Areas in Selected Low- and Middle-Income Countries.

*Source*: Authors’ analysis using data from the World Bank’s Service Delivery Indicators Education Survey.*Note*: We calculated teacher absenteeism rates from the attendance records of the second unannounced visit in the survey. Averages are first computed at the school-level and aggregated at the country level using school-level weights. Differences in rural and urban/semi-urban rates are tested using bivariate regressions with school-level weights and robust standard errors and are presented here with statistical significance: * significant at 10%; ** significant at 5%; *** significant at 1%.

Teacher absenteeism, on the other hand, is higher in rural areas in every country in our sample except Tanzania (Fig. 2), and the differences are sizeable: around ten percentage points or higher in five out of eight countries. Absenteeism is not a measure of quality in the same way that teacher competencies are: it is likely a result of teacher management practices and of the fact that teachers in rural posts may travel more frequently to spend time with families that have not moved to rural areas (e.g., on Fridays and Mondays). However, from the perspective of a student, this is an indicator of quality, since any absenteeism means that students are not learning from teachers. Consistent with this, students in rural areas tend to perform worse on exams (Cattaneo et al., 2022). While this may be in part a function of all the factors listed above, it may contribute to a vicious cycle in which teachers—and especially highly skilled teachers—may be reluctant to transfer to schools with high concentrations of struggling students.

## 3 Policy options for hiring and retaining teachers in hard-to-staff schools

Education systems have a wide array of policies that can potentially narrow the staffing gap between hard-to-staff schools (in rural or otherwise high-poverty areas) and other schools. Since hard-to-staff schools tend to lack amenities that other schools have, education systems must provide a compensating wage differential or other incentive to reaching candidates in order to attract them to such schools. This wage differential may come in the form of monetary inducements or other benefits.^4^ Table 2 lists these policies, which fall into three broad categories: material benefits, professional benefits, and others. Many countries simultaneously implement a package of these policies to address different co-existing challenges. In some cases, knowledge of existing programs is low among teachers and so governments complement programs with enhanced publicity campaigns (Ajzenman et al., 2021a; Elacqua et al., 2022; Rosa, 2019). Below, we briefly describe each policy and provide specific examples of either current or historical policies in specific countries. The country examples below all come from Pugatch and Schroeder (2014) unless explicitly sourced from elsewhere, with many of Pugatch and Schroeder's examples having been drawn from McEwan (1999).^5^

**Table 2:** Policy levers for attracting and retaining teachers in hard-to-staff schools in low- and middle-income countries.

Material benefits	Professional benefits	Other policies
• Financial incentives / hardship pay • Housing • Moving expenses • Travel allowances	• Accelerated path to promotion • Additional training • Reduced hiring requirements • Study leave with pay	• Mandatory rotations • Local hiring drives • Behavioral nudges
Complementary policy: Providing information on policies		

*Source:* This list draws on Elacqua et al. (2018); McEwan (1999), and Pugatch and Schroeder (2014).

*Notes:* This is a non-exhaustive list.

Among material benefits, probably the most common program is a financial incentive, commonly an increased salary for as long as the teacher is stationed at a school that qualifies as a “hardship post” (Elacqua et al., 2018). These range from a monthly bonus as a percentage of the base pay (in Honduras, Lesotho, and the Philippines, among many others), to a rise of a couple of steps in the standard pay scale (in Jamaica), to a pay raise as a reward after a certain number of years of rural service (in Venezuela). Some countries have multiple levels of hardship bonuses, depending on the remoteness of the posting (in Mozambique and Zambia). In high-income countries, student loan forgiveness is another material benefit that some governments offer (Feng & Sass, 2018).

Other education systems provide non-pecuniary material benefits, such as support for housing for teachers, since housing markets may be less liquid in remote areas. That may include direct provision of housing (in Iraq and Pakistan), a subsidy for housing (in Senegal and Sierra Leone), or loans for housing (in Syria). Beyond housing, countries less frequently provide other material benefits, such as moving expenses (in Libya) or travel allowances to allow teachers to purchase consumer goods (in Guyana).

A second class of benefits includes professional benefits, such that teachers may advance their career more rapidly by accepting a hardship posting. This might include accelerated promotion opportunities (in Egypt, Guyana, Honduras, and Zambia), special training for rural service (in Bangladesh, Colombia, and Nicaragua), or an accelerated clock towards retirement (Costa Rica). The flip side of professional benefits is to reduce professional qualification requirements, as the government has done in the past for teachers in rural schools in Cambodia.

Mandatory rotations are another policy used in many countries, where teachers are required to spend a certain amount of time in hardship schools. Syria at one point had mandatory rural service at the start of the teaching career (McEwan, 1999), and China has a program that required urban teachers to work for a certain period in rural schools (Liao et al., 2019). Many countries have also implemented efforts to hire teachers locally, presuming that teachers from the local area may be less averse to a hardship posting. Ghana implemented a program like this by encouraging districts to provide scholarships for students originally from those districts to train as teachers (Cobbold, 2006). More recently, Ecuador has experimented with behavioral nudges—e.g., moving hardship schools higher on the list of schools that candidates can choose to apply to Ajzenman et al. (2021b).

## 4 Methods for this systematic review

**Fig. 3:**
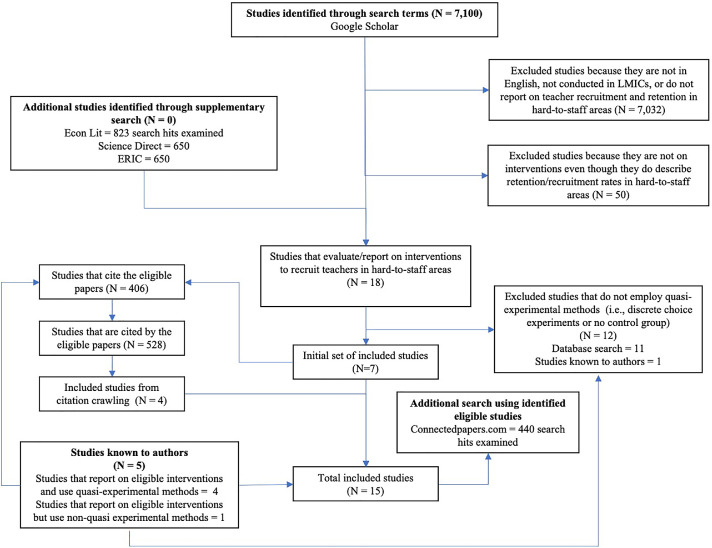
Consort Diagram of the Search and Review Process to Identify Experimental and Quasi-Experimental Studies.*Source:* Authors’ illustration based on this study’s search and review strategy.

Our search strategy focused on experimental or quasi-experimental evaluations of government interventions to recruit and retain teachers in hard-to-staff schools such as those in rural and remote areas, lower income neighborhoods, or otherwise disadvantaged communities in low- and middle-income countries. We did not impose a limit on publication dates for the search. We searched for papers written in English and published in peer-reviewed journals, working paper series, and academic conferences. The search was initially conducted between June and August 2021 and again in October 2022 to update the database. Fig. 3 provides an overview of our search process and the results of the combined searches.

We used Google Scholar as the primary search database and used the following search terms: (“teacher” OR “teaching” OR “school”) AND (“hard-to-staff” OR “disadvantaged” OR “remote” OR “rural” OR “high poverty” OR “underprivileged’ OR “hardship”) AND (“staff” OR “staffing” OR “incentive” OR “allowance” OR “bonus” OR “higher salary” OR “higher pay” OR “housing” OR “transportation” OR “promotion” OR “retirement” OR “training”) and “country X,” where “country X” is substituted by each low- and middle-income country name as classified by the World Bank in 2020 (World Bank, 2022). We include papers regardless of publication status (e.g., working papers and conference papers as well as journal publications) to limit publication bias. We evaluated the title, abstract, and the full text of the papers as needed to review eligibility. Since each unique search often returned hits in the thousands, we narrowed the screening by reviewing the first 50 hits from each search, sorted by relevance. In total, we reviewed 7,100 hits from Google Scholar. The majority of the studies were excluded (7,031 studies) because they were either not conducted in low- and middle-income countries, not designed to recruit teachers in hard-to-staff areas, did not report relevant outcomes such as teacher recruitment and retention or related student performance, were not government implemented, or were not in English. Another 50 studies were excluded because they did not evaluate interventions, even though they did describe retention/recruitment rates in hard-to-staff areas. Another 11 studies that did report on the topic of recruiting teachers in hard-to-staff areas in low- and middle-income countries were excluded from the primary sample because they did not employ experimental or quasi-experimental methods—i.e., they used discrete choice experiments or had no control group.^6^ This stage of the screening yielded 7 eligible studies.

Recognizing the limitations of Google Scholar as a search system (Gusenbauer & Haddaway, 2020), we supplemented our primary search with additional, limited searches in EconLit (823 search hits), ScienceDirect (650 search hits) and the Education Resources Information Center (ERIC) (650 search hits) to ensure we had not missed relevant studies. We used the same search terms but with (a) no geographical identifier, (b) a regional identifier such as “…AND Africa” instead of the country identifier, and (c) the country name for the most populous country in each region (e.g., “… AND Nigeria”), and evaluating the first 50 hits of each unique search or all the hits, whichever number was smaller. We found several studies that were already in the database but no additional papers.

For the next stage of the search, we reviewed the papers that cited the studies that passed our eligibility criteria and studies that were cited by the eligible studies, repeating the citation crawl for any new eligible study. In total, we reviewed 406 studies that cited our eligible studies and 528 studies that were cited by our eligible studies. This stage added four eligible studies to the list. Next, we reviewed five studies that were known to the authors but did not come up in the search. Four of those five studies passed all of the eligibility criteria. The remaining study was a discrete choice experiment: this was added to the list of studies that report on the topic of recruiting teachers in hard-to-staff areas but whose methodology did not pass the eligibility criteria. (This brings the total of such studies—on our review's topic but not using the evaluation methods we focus on—to twelve studies. We list these studies in Table A.4.) Finally, we ran all the eligible studies through Connected Papers, a search tool that returns a list of other papers with bibliographies that are highly similar to the bibliography of the original paper (Eitan et al., 2022). We reviewed 440 papers and found several studies that were already in the database but no additional papers.

In total, we identified 15 studies that pass our eligibility criteria (Table 3). All of those studies are from either Latin America (Bolivia, Brazil, Chile, Ecuador, Peru, and Uruguay), Sub-Saharan Africa (the Gambia, Tanzania, and Zambia) or South Asia (India). Chile and Uruguay are high-income countries today, but they were classified as middle-income through 2012 (World Bank, 2013), and most of the studies draw on data from years before that. Of the thirteen total quasi-experimental evaluations, ten use regression discontinuity designs, one uses a difference-in-differences strategy, and two use matching strategies.^7^ We include a discussion of strengths and weaknesses of the different strategies in our narrative of the results.

**Table 3:** List of 15 Included Studies of Interventions.

**Study**	**Type of program**	**Type of schools or teachers targeted**	**Target outcome**	**Country**	**Year of intervention evaluated**	**Identification strategy**
Ajzenman et al. (2021a)	Informational interventions emphasizing either financial incentives or altruistic motivations	Disadvantaged schools which are “typically low-performing and understaffed”	Recruitment	Peru	The informational interventions were implemented in 2019.	Randomized controlled trial
Ajzenman et al. (2021b)	Behavioral nudge (listing hard-to-staff schools first in job application platform)	Schools that are “typically located in more remote and vulnerable areas that normally have greater difficulty attracting teachers”	Recruitment	Ecuador	Platform change was implemented in 2019.	Randomized controlled trial
Bobba et al. (2022)	Financial incentive (government-sponsored wage bonus)	Schools in rural areas	Recruitment and retention	Peru	Policy was first implemented in 2014 with increase in bonuses in 2015.	Regression discontinuity
Cabrera and Webbink (2020)	Financial incentive (increase in base salary)	Teachers working in poor neighborhoods	Recruitment and retention	Uruguay	Program was launched in 1995 and updated in 2005 to use a poverty index cut-off as eligibility criteria.	Regression discontinuity
Camelo and Ponczek (2021)	Financial incentive (wage premium for teachers at disadvantaged schools)	Schools in poor urban areas defined by a socioeconomic index	Retention	Brazil	Program was launched in 2008.	Regression discontinuity
Castro and Esposito (2022)	Financial incentive (rural bonuses)	Rural schools chosen “based on the distance from the closest provincial capital and population”	Recruitment and retention	Peru	The program was started in 1990 and revamped in 2014 to increase bonuses and include more schools.	Regression discontinuity
Chelwa et al. (2019)	Financial incentive (rural hardship allowance)	“schools outside a given radius from district centers”	Recruitment and retention	Zambia	Allowance was implemented in the 1990s, revamped in 2008 (to become 20% salary increase), and implementation rules were changed in 2010.	Regression discontinuity
Chin (2005))	Other intervention (government recruitment drive for additional teachers)	Primary schools with only one teacher	Recruitment	India	Launched in 1987, the program served all originally targeted schools by 1994.	Difference-in-differences
Elacqua et al. (2022)	Financial incentive (salary bonus)	Schools where “60 percent or more of the students enrolled were from families with low socioeconomic status”	Recruitment and retention	Chile	Program under evaluation was implemented between 2012 and 2015.	Regression discontinuity
Hinze-Pifer and Méndez (2016)	Financial incentive (additional bonus for teachers in disadvantaged schools)	Schools that have high share of poor students, more rural, or in neighborhoods with high crime or poverty rate	Recruitment and retention	Chile	Program was originally established in 1996.	Regression discontinuity
Pugatch and Schroeder (2014, 2018)	Financial incentive (hardship allowance for school teachers in remote locations)	Remote schools defined by distance from the capital.	Recruitment and retention	Gambia	Allowance policy was adopted in 2005.	Regression discontinuity
Rosa (2019)	Financial incentive (wage premium for teachers in more remote schools)	Schools farther from downtown	Recruitment	Brazil	Classification of neighborhoods and corresponding wage premium was set up in 1991.	Regression discontinuity
Swai (2013)	Financial incentive (cash bonus to attract teachers in the region)	Schools in the remote Rukwa region	Recruitment and retention	Tanzania	Policy was initially implemented in 2004.	Matching
Urquiola and Vegas (2005)	Financial incentive (salary bonus to rural teachers)	Schools in areas classified as rural by the Ministry of Education	Recruitment and retention	Bolivia	Bonus based on geography is based on different incentive programs. The bonus reported in the study reflect 2002 salary levels.	Matching

Note: See Appendix Tables A.5 and A.8 for additional details on the interventions, research designs, evaluation timeframes, samples, and outcomes.

In this review, we principally report the results in narrative form rather than as a meta-analysis because the studies vary widely in their outcomes, making meta-analysis more difficult to interpret. To supplement our narrative analysis, we also report vote counts (i.e., the distribution of positive and negative results across studies), which should be viewed as suggestive only, given the limitations of that method (Higgins & Green, 2011). One limitation of vote counting is that it ignores crucial data such as sample size, statistical precision (beyond significance cut-offs), and effect size. As a result, small but significant effects may be overweighted relative to large but just barely insignificant effects, even though the latter may have greater policy implications (Evans & Popova, 2016). One partial solution is to focus on the distribution of positive versus negative results rather than on statistical significance (Higgins & Green, 2011; McKenzie & Brennan, 2022). Thus, we incorporate vote counting only as a complement to our narrative synthesis.

## 5 Results

### 5.1 Distribution of evidence across policies

One of the key findings from this search is how limited the empirical evidence is for most policies to attract and retain teachers in hard-to-staff schools. Table 4 lists the classes of policies, the amount of experimental or quasi-experimental evidence we find of the impact of each type of policy on either teacher or student outcomes, and a brief summary of the evidence. (In the course of our search, we identified non-experimental studies that seek to measure preferences across options or to describe programs; we discuss those in Section 5.5.) Twelve of the impact studies we identified are quasi-experimental evaluations of various forms of financial incentives for teachers in hard-to-staff schools, either rural areas or high-poverty schools, one is a quasi-experimental evaluation of a recruitment drive for short-staffed schools, and the other two are experimental evaluations of interventions to encourage more teachers to list hard-to-staff schools among their choices for potential assignment. Most of the financial incentive programs have positive impacts on some aspect of teacher supply. The predominance of financial incentives among evaluated programs is consistent with the pattern in a recent systematic review focused on high-income countries, where all the well-identified studies were of financial incentives (See et al., 2020). This means that of the 10 policies that we list in Table 4, we lack experimental or quasi-experimental evidence on 7 of them. In other words, there is much more unknown than there is known in terms of the impact of these policies on teacher supply, teacher quality, and student learning outcomes.

**Table 4:** Causal evidence of impact of different policy options on teacher supply or student outcomes.

Policy	Studies that report teacher or student outcomes		Summary of evidence
	Experimental (2 studies)	Quasi-experimental (13 studies)	
*Material benefits*			
Financial incentives/hardship pay	0 studies	12 studies (Bobba et al. 2022; Cabrera and Webbink 2020; Camelo and Ponczek 2021; Castro and Esposito 2022; Chelwa et al. 2019; Elacqua et al. 2022. Hinze-Pifer and Méndez 2016; Pugatch and Schroeder 2014 and 2018; Rosa 2019; Swai 2013; Urquiola and Vegas 2005)	• Most financial incentive programs have positive impacts on teacher outcomes, including placement in hard-to-staff schools and reducing turnover. • Many also have positive impacts on student learning for at least some groups of students, although these outcomes are measured less consistently. • Many of the programs face implementation challenges, including that teachers are often unaware of the programs.
Housing	0 studies	0 studies	No causal evidence.
Moving expenses	0 studies	0 studies	No causal evidence.
Travel allowance	0 studies	0 studies	No causal evidence.
*Professional benefits*			
Accelerated path to promotion	0 studies	0 studies	No causal evidence.
Additional training	0 studies	0 studies	No causal evidence.
Reduced hiring requirements	0 studies	0 studies	No causal evidence.
Study leave with pay	0 studies	0 studies	No causal evidence.
*Other policies*			
Mandatory rotations	0 studies	0 studies	No causal evidence.
Local hiring drives	0 studies	1 study (Chin 2005)	• With just one study (in India), a federally funded program had little impact on teacher supply due to compensatory behaviors by state governments.
Behavioral nudges	2 studies (Ajzenman et al. 2021a; Ajzenman et al. 2021b)	0 studies	• Providing information to teachers about the positive difference they could make as teachers led to higher placements in hard-to-staff schools in Peru. • Increasing the salience of hard-to-staff schools as employment options in Ecuador increased placements. • These low-cost options have modestly size impacts but are likely to be cost-effective.

*Source*: The policies are based on Table 2. The number of studies for each policy is based on studies we found during the search documented in Fig. 3.

*Notes*: Experimental studies include those in which assignment is random. Quasi-experimental studies include those that identify the impact of the benefit using methods such as difference-in-differences, regression discontinuity, or matching. (See footnote 5 in the article for our motivation for including matching studies among the quasi-experimental studies.) One study may evaluate interventions with multiple components. Because the focus of this review is government-implemented programs, we do not include here non-government organization (NGO) implemented programs.

### 5.2 Impacts on the teacher workforce

Twelve of the fifteen studies in our sample use quasi-experimental designs to identify the impact of financial incentives on teacher outcomes (and in most cases, on student outcomes as well). Of these, one study is designed to recruit teachers during the hiring process to hard-to-staff schools (Rosa, 2019), another study is to improve retention of existing teachers (Camelo & Ponczek, 2021), while the remaining studies are on interventions aimed at improving both recruitment and retention. The three non-financial incentive studies are all aimed at attracting teachers during the hiring process. One study evaluates a government-sponsored recruitment drive for additional teachers in short-staffed areas, and two other studies explored alternative strategies to boost teacher placement in hard-to-staff schools. We summarize all teacher outcomes in each of the fifteen studies in detail in Table A.5. On the whole, across all teacher outcomes—placement, turnover, competence, working hours, holding a second job, and expressed preferences—the vast majority (60 percent) are positive (Table A.6). None of the outcomes have fewer than 69 percent of results positive, except for the outcomes related to working hours reported by just two studies. Teacher turnover stands out with 89 percent positive. If these interventions were ineffective at changing teacher outcomes, we would expect an equal distribution of positive and negative impacts.

#### 5.2.1 Financial incentives

Almost all of the twelve studies that measure the impact of financial incentives use regression discontinuity designs, comparing schools that barely pass the eligibility criteria for teacher financial incentives to those that fall just short of eligibility. Most focus on efforts to increase the quantity of teachers in hard-to-staff schools. In Peru, the government implemented a wage increase for teachers in rural schools at three different levels: the highest increase, for teachers in “extremely rural” schools was equal to between 20 and 30 percent of the earnings of permanent teachers (Bobba et al., 2022). In Peru's centralized teacher allocation system, contract teachers with the highest test scores get first choices for open positions. (Assignment for permanent teachers includes test scores but also other factors.) While the wage increase had no impact on vacancies filled, it did lead to the placement of contract teachers with scores that were 0.45 standard deviations higher in hard-to-staff schools. Subsequent impacts on student learning were concentrated among schools that had openings for teachers, suggesting that the wage increases had no impact on the effort of incumbent teachers, consistent with existing data on unconditional wage increases (De Ree et al., 2018). The authors go on to simulate a model suggesting that a more precise, data-driven policy that incorporates teacher preferences could—at no additional cost—both reduce vacancies and increase teacher quality substantively (Bobba et al., 2022).

Similar rural bonuses have been implemented in Zambia and the Gambia. Chelwa et al. (2019) report on a rural hardship allowance in Zambia which increased salaries by 20 percent for teachers in schools outside a pre-specified distance to district city centers (computed using global positioning system—or GPS—data). Schools could appeal their eligibility if natural barriers such as mountains made the actual travel distance longer than the GPS distance. Implementation changes and errors—subsequent reclassification of schools and a teacher-payroll mismatch where teachers were still listed in schools where they no longer taught—meant that passing the GPS-eligibility criteria increased the share of teachers receiving the allowance by only 40 percent. (Full compliance and accurate payroll would make this 100 percent.) Limiting the analysis to provinces with higher compliance as verified by telephone survey, the authors find positive but weak evidence that the program increased the quantity of teachers (by 10 percent) and teacher tenure (by between 0.5 to 0.8 years). Impacts may have been muted further due to payment delays (as reported by teachers), a documented challenge in many countries (Evans & Yuan, 2018).

In the Gambia, Pugatch and Schroeder (2014) evaluate the rural hardship allowance and find stronger but similarly positive results. The Gambian rural allowance awarded a salary premium between 30 and 40 percent to teachers in primary schools located 3 kms or more from a main road. (Hardship schools in regions that were farther from the capital of Gambia received higher bonuses.) The program increased the share of qualified teachers by 10 percentage points and reduced the ratio of students to qualified teachers by 61 percent of the mean (27 students). Teachers not only chose hardship areas over non-hardship areas, but they were also responsive to the higher incentive provided in the most remote regions: increasing the hardship allowance by 10 percentage points increased share of qualified teachers by 2.8 percentage points.

Rosa (2019) reports on an initiative in the city of São Paulo, Brazil to provide a wage premium for teachers in schools that are farther from downtown. The premium, evaluated using data from 2010, was based on salary values in 1991 (30 to 50 percent of teacher wages at that time) and was not corrected for inflation in succeeding years, leading to the actual premium being just 5 to 7 percent of 2010 wages when data for evaluation was collected. In addition, assignment of which neighborhoods qualified for the wage premium had also not changed since the 1990s, creating adjacent neighborhoods with similar socioeconomic indicators but in different wage-premium zones. The incentive had no significant effect on teachers’ preferences towards hard-to-staff schools, potentially because the incentive was too small to change preferences or because the incentive did not appear in official hiring documents, such that teachers may not have even realized the policy was in place when making employment decisions.

Many of these hardship allowance policies use eligibility criteria based on location (distance from provincial capital or road). But some hard-to-staff schools face other vulnerabilities that are not exclusively tied with geography. Cabrera and Webbink (2020) evaluate an Uruguay program (*Contexto Socio Cultural Crítico*) program that provides up to a 26 percent increase in base salary for teachers who choose to transfer or who are already working in schools with a high poverty index (a composite score based on various student characteristics). The program did attract more experienced teachers to schools in target neighborhoods, leading to an increase in average teacher experience of around three to seven years in beneficiary schools. The program also increased how long teachers stayed in their current school by one to two years (compared to a pre-treatment average stay of five years). In Brazil, Camelo and Ponczek (2021) evaluate the national program Bonus for Place of Work (*Adicional por Local de Exercício*) salary incentive for teachers working in disadvantaged schools, in this case defined by the municipal-level socioeconomic index of household income, characteristics of the household head and the family composition. Staff (teachers, principals, and support staff) received an additional compensation of between 24 and 34 percent of their base salary, depending on their job position and seniority. The program reduced teacher turnover by 5 percentage points (about 10 percent over pre-treatment average) over the four years succeeding the introduction of the policy in 2008.

Hinze-Pifer and Méndez (2016) evaluate an incentive program in Chile called the Difficult Conditions Bonus (*Asignación de Desempeño en Condiciones Difíciles*). This program awarded incentives of between 4 percent and 30 percent of salary based solely on choosing hardship schools. Eligibility was measured by an index of disadvantages including share of low-income students, distance from a large city, and public transit access. The more disadvantaged schools received a higher bonus, with all teachers in an eligible school receiving the same bonus regardless of performance. In addition, schools must apply to be eligible for the program every two years. The program did not significantly affect teacher retention and—in the face of opaque bonus calculation and unpredictable delivery—it actually led to fewer contract hours worked and teaching hours per student. These teachers were paid an hourly wage, so the higher pay may have enabled them to work fewer hours in difficult-to-teach circumstances.

To round out studies explicitly focused on increasing the quantity of teachers in hard-to-staff schools, Urquiola and Vegas (2005) and Swai (2013) use matching designs in Bolivia and Tanzania. Matching designs tend to be less credible than discontinuity designs because observers usually have less confidence that the treatment and comparison groups are comparable (besides the effect of treatment), particularly since treatment may be assigned based on both observed and unobserved characteristics. So we put less weight on these studies, but we include them for readers’ awareness and in the interest of expanding geographic coverage. Bolivia has had several programs that provide additional salary to teachers based on geography—including a financial incentive for teachers in poor and rural regions, a bonus for “inaccessible areas,” and a bonus for teaching in schools within 50kms of international borders—which together provided up to 12.5 percent of the teachers total wage bill (Urquiola & Vegas, 2005). The different potential geographically determined bonuses, combined with the Ministry of Education's hesitation to remove the bonus of teachers who teach in schools that have been reclassified from rural to urban in previous years (for fear of union opposition) means that teachers may have similar training and experience and work in geographically similar schools but have different salary levels. Urquiola and Vegas (2005) match schools classified as urban and rural but all located within three large cities. So-called rural schools in this case used to be rural but as cities have grown, they have fallen within city borders, albeit further from the center. While they do not report recruitment or retention outcomes, they find that teachers in schools classified as “rural” work fewer hours less than teachers in schools classified as “urban” (a difference of about one third of a standard deviation) and are 16 percentage points less likely to hold a second job. Because of the matching design, it is difficult to interpret how much of the results may be due to selection. However, the reduction in work hours is consistent with the finding of Hinze-Pifer and Méndez (2016) in Chile.

Swai (2013) uses a matching design in Tanzania and reports on the Rukwa Civil Servant Facilitation Fund in Tanzania, which provided a signing bonus to secondary school teachers recruited in the rural region equivalent to at least one month of take-home pay in addition to accommodations and other inputs. The evaluation did not find statistically different retention rates between schools in the Rukwa region and the neighboring Kigoma region (which served as one comparison group). Within the Rukwa region, there was no significant difference in the retention rate between teachers recruited through the incentive system and teachers recruited via traditional means (the other comparison group).

As demonstrated above, teacher quality is a further challenge in hard-to-staff areas. Elacqua et al. (2022) report on the Pedagogical Excellence Assignment program in Chile, which awarded a monetary incentive with an additional bonus of 40 percent of the base value of the award (equivalent to 16 percent of a teacher's average annual salary) to teachers working in schools where at least 60 percent of students were considered low-income.^8^ This is the only program in our sample explicitly designed to improve quality. The bonus applied to both newly hired teachers and teachers that transferred to disadvantaged schools; consequently, teachers lost the bonus when they moved out of disadvantaged schools. Winning the award increased the probability that teachers who were already working in disadvantaged schools continued to do so two years after the award (by 17 to 21 percentage points). On the other hand, winning increased the probability that teachers at more advantaged schools at baseline would still be working in similarly advantaged schools two years after (by 36 to 44 percentage points), suggesting that many teachers used the award as a quality signal to stay or move into more desirable posts.

One limitation of these regression discontinuity designs is the potential for spillovers: if there is a clear boundary between advantaged and disadvantaged schools, then a bonus for teachers in disadvantaged schools may draw teachers from just across the boundary (the control group in discontinuity designs), leading the impact of the bonus to be overstated. This overstatement is due to what might be thought of as “double counting.” Schools on the advantaged side of the boundary grow slightly worse off because teachers leave, and schools on the disadvantaged side grow slightly better off because the same teachers arrive. Both the improvement and the deterioration (despite being part of the same teacher movement) are counted.

One estimate of the magnitude of that spillover effect comes from an evaluation of the Peruvian rural allowance, which provides a bonus of about 26 percent of a teacher's starting salary. Castro and Esposito (2022) compare schools in the most remote category (with the highest bonus), reserved for schools in communities that are more than 120 minutes from a provincial capital and have fewer than 500 inhabitants, to similar rural communities on the other side of the threshold, which receive a slightly lower bonus. Three years after the incentive was launched, the differential recruitment bonus reduced attrition by between 1.5 and 4.9 percentage points and increased the proportion of vacancies filled by between 1.6 and 3.4 percentage points, with the larger effects in the most distant schools. However, in control schools that are less than 30 min away from the treatment schools, the proportion of teacher vacancies filled dropped by between 2.5 and 3.1 percentage points.

This substitution between schools that are near each other has two implications. First, it means that bonuses may lead to improvement in some schools at the expense of schools that are almost as hard-to-staff themselves; indeed, teachers already in semi-rural areas may mind transferring to more rural schools less. If teachers are less effective in their first year or two in a new school, then the net effect may even be negative. Second, it means that the estimated impact of bonuses for programs that do not account for spillovers may be overstated. For comparison, the other study on Peruvian rural incentives by Bobba et al. (2022) does not account for spillovers and reports more positive outcomes on teacher recruitment. That paper also uses data from a longer timeframe, extending to more recent data (from 2015 to 2018) than the Castro and Esposito (2022) study (data from 2016).

While most studies do not engage the potential impact of spillovers, at least two do. Cabrera and Webbink (2020) highlight this potential negative externality (from hiring experienced teachers away from other schools) but do not have available data to verify this. Pugatch and Schroeder (2014) report back-of-the-envelope calculations to estimate relative supply and demand of teachers, which suggests that the gains in qualified teachers from the Gambian rural allowance are not just from teachers switching from non-hardship to hardship schools. The degree to which incentives draw teachers from schools that are only slightly less disadvantaged is crucial to understanding the impact of these programs going forward.

#### 5.2.2 Other interventions

Other interventions do not explicitly involve financial incentives to teachers. A study in India used a difference-in-differences strategy to evaluate a national recruitment drive implemented between 1987 and 1994 for teachers to be deployed exclusively to one-teacher primary schools, called Operation Blackboard (Chin, 2005). The study compared states with many primary schools with only one teacher (which received a higher intensity of the program) to states with few primary schools with only one teacher (lower intensity of the program). In this intervention, the central government took responsibility for paying the salary of these new teachers in the initial few years, after which the state government took over and paid the teachers’ salaries for the subsequent years. The program aimed to recruit and pay for 140,000 new teachers (around 8 percent of pre-program teacher supply in the country). However, only between a quarter to a half of these teachers were placed in target schools. The average number of teachers per primary school and the pupil to teacher ratio also did not increase, suggesting that the nationally funded program merely created incentives for state governments to redistribute existing teachers from larger schools to smaller schools, slow down their own hiring efforts, or likely both.

Another class of interventions provides information to teachers to increase their likelihood of applying to work in hard-to-staff schools. These behavioral interventions are promising largely because they are much cheaper to implement than an increase in pay. Ajzenman et al. (2021a) tested two interventions to increase applications of newly accredited teachers to hard-to-staff schools in Peru. The first emphasized the altruistic nature of teaching: candidates were invited to reflect on their reasons for becoming a teacher, received text messages reminding them of this role of teachers (e.g., “thank you for being an agent of social change”), and saw pop-ups with similar messages on the online application platform. The second intervention used the same three tools, but rather than focusing on altruistic motives, it focused on the financial benefits (including an already existing financial incentive) and career path advantages associated with working in disadvantaged schools. A third set of schools received placebo messages with general application information. The two interventions had similarly sized impacts: teachers in the two treatment groups increased the proportion of disadvantaged schools in their set of application schools by about 2 percentage points (relative to 46 percent at baseline). The effect is driven by male candidates. Perhaps unsurprisingly, the intervention that focused on financial benefits was more effective among candidates who performed worse on the test (and were also likely lower income), whereas the altruism-focused intervention appealed to higher scoring teachers. Since higher scoring teachers were more likely to get their preferred schools, only the altruism-focused intervention ultimately resulted in more teachers assigned to disadvantaged schools: male teachers in that group were 3.4 percentage points more likely to be assigned to a hard-to-staff school, and male teachers who scored above median on the qualifying exam were 5.2 percentage points more likely.

In Ecuador, teachers similarly pass a series of exams and then use an online platform to apply for school vacancies. In this context, researchers tried an even lighter touch intervention (Ajzenman et al., 2021b). In the treatment group, hard-to-staff schools were listed first in the online platform, whereas in the control group, schools were listed alphabetically. For both groups, hard-to-staff schools were identified with an icon on the list. Candidates in the treatment group were about 5 percentage points more likely to rank a hard-to-staff school first, and they were about 3 percentage points more likely to accept a position at a hard-to-staff school (relative to a 27 percent likelihood in the control group). This impressive result from simply re-ordering school names was likely driven by simple choice overload: with lots of options, it was easier to pick the first ones. How impacts are sustained over time is of interest with all intervention designs, and this one is no exception, as new teachers may be more explicitly aware of the nudge and then incorporate that knowledge into their selection behavior over time.

These studies provide promising evidence for this kind of behavioral intervention, as either a complement (in Peru) or a substitute (in Ecuador) to financial incentives. While outcomes across studies are mostly not strictly comparable, the ultimate effect of these behavioral interventions is likely to be smaller than most of the financial interventions (i.e., the biggest effects of the behavioral interventions are on teachers putting hard-to-staff schools on their choice lists), although they may be more cost-effective since they are extremely cheap. Thus, they are unlikely to fully close gaps between hard-to-staff schools and other schools, but they are a valuable, innovative tool in the policymaker's toolkit.

#### 5.2.3 Evidence of heterogeneity

Although the number of interventions for which we have evidence is very limited, we examine the association between the size of a financial incentive and the likelihood of a positive or positive and significant impact (Table A.7). If we exclude financial incentive programs with mixed results, we have just ten interventions remaining. We see a positive, significant association between the size of the incentive and the likelihood of a positive, significant impact (Fig. A.3), and a positive but insignificant association between the size of the incentive and the likelihood of a positive impact (thus stepping away from the challenge of power in individual studies). Thus, there is limited, suggestive evidence that larger incentives may have more positive impacts, as one might expect.

We also examine the association between the degree of rurality in the country and the effectiveness of the programs and find no significant association and no consistent sign (Table A.7). Of course, the effectiveness of incentive programs may depend on many more characteristics of the education system than the mere degree of rurality; but with such a small sample of interventions, meta-regression capturing several aspects of the education system would be underpowered.

### 5.3 Impacts on student outcomes

One of the primary objectives (if not the primary objective) of an education system is to increase student learning (World Bank, 2018). Thus, increasing the quantity or even the quality of teachers in hard-to-staff schools is primarily a means to an end, with the desired outcome being improved student outcomes, whether those be access or achievement. Of the 15 studies, while all but one report teacher-related outcomes, only 9 report student-related outcomes.^9^ All student-related outcomes are summarized in Table A.8. Just as with teacher outcomes, the vast majority of student-related outcomes are positive (77 percent), with a higher percentage for student attendance (92 percent) than for student achievement (72 percent) (Table A.9). There is a similar pattern of statistical significance for student versus teacher outcomes for studies that report both sets of outcomes (comparing Tables A.6 and A.9), and studies that include student outcomes have a similar distribution of positive (and significant positive) impacts on teachers as studies that do not include student outcomes. Across all studies that report student outcomes, 74 percent of teacher outcomes are positive, and 37 percent are positive and statistically significant. Across studies that do not report student outcomes, 68 percent are positive, and 45 percent are positive and statistically significant. In other words, with our limited sample, we do not have reason to believe that the distribution of reported student outcomes is biased.

Despite this broad pattern of positive, significant impacts, there is heterogeneity across studies: one of the interventions improved student test scores across the board, some have heterogenous impacts, and some did not affect student outcomes at all. In Peru, students in target rural schools improved their test scores in Spanish and math with effect sizes of 0.30 to 0.35 standard deviations (Bobba et al., 2022). Consistent with the finding that the program improved the quality of recruited contract teachers, the effect on student test scores was bigger in schools with short-term teacher vacancies (0.32 standard deviations in Spanish and 0.47 standard deviations in math). (Short-term vacancies are open for one academic year, with contracts renewable for up to one more year subject to administrative approval.)

In Brazil, an incentive program had no effect on average student test scores, but it did reduce the proportion of low-performers in math (by 6.8 percentage points, or an 11.3 percent absolute reduction) and reading (by 5.4 percentage points, or a 17.4 percent absolute reduction) (Camelo & Ponczek, 2021). In the Gambia, the rural allowance had no effect on average test scores, but it did improve student outcomes for the subset of students with higher socioeconomic status (by about 0.40 standard deviation in math and English test scores) (Pugatch & Schroeder, 2018). Girls and boys were equally likely to benefit from the program. The rural bonus program in Zambia also had no effect on average student performance but did show some positive effect for boys—they were 2 percentage points more likely to have a score that qualifies for the highest category in the national exams—but not for girls (Chelwa, Pellicer & Maboshe, 2019). The recruitment drive for additional teachers in India improved primary completion rates, especially for girls (by between 0.91 to 1.61 percentage points for each teacher recruited per 1000 children) and for girls in households at the bottom expenditure quartile (by between 2.23 to 2.95 percentage points). The impact on primary completion rates for boys was also significantly positive (between 1.00 to 1.64 percentage points) but only for some model specifications.

The incentive program in Uruguay did not improve student test scores or grade-retention, or drop-out, although it reduced insufficient student attendance by 15.3 percentage points in one specification (but not in others) (Cabrera & Webbink, 2020). Similarly, the incentive program in Chile did not improve test scores for 4th Grade students, either for math or reading, in year 1 or year 2 of the policy (Hinze-Pifer & Méndez, 2016). Finally, the rural pay differential in Bolivia is not systematically related with student test scores or grade repetition, but is slightly correlated with both higher pass rates and higher drop-out rates (between 0.6 to 1.4 percent change, significant only in some specifications) (Urquiola & Vegas, 2005).

Given potential spill-over effects on teacher recruitment and retention, we might expect to see spill-over effects on student outcomes as well. Surprisingly, the rural bonus in Peru—where the authors explicitly measured spillovers—did not affect student scores in schools with the bonus, but students in control schools less than 30 min away from beneficiary schools experienced up to 0.30 standard deviation gains in both reading and math scores, despite having more teacher vacancies go unfilled (Castro & Esposito, 2022). This may imply that the rural bonus recruited lower skilled teachers away from neighboring control schools to bonus schools, leading to higher average teacher quality (if lower quantity) in control schools.^10^ Neither of the behavioral interventions (Ajzenman et al., 2021a; Ajzenman et al., 2021b) nor the three other financial incentive interventions (Elacqua et al., 2022; Rosa, 2019; Swai, 2013) report student outcomes.

### 5.4 Women teachers and hard-to-staff schools

There is evidence that teachers who are women can have positive impacts both on student learning and on girls’ aspirations, particularly in secondary school (Eble & Hu, 2020; Lim & Meer, 2020). Yet parity in teacher gender ratios can be harder to achieve in more remote schools. In Peru, teachers who are women were more likely to choose schools in urban areas and closer to where they attended their initial teacher training program (Bertoni et al., 2021). In the Gambia, the share of women teachers in schools that qualified for hardship allowances (classified according to distance from the main road) was 10 percentage points lower than the country average (Pugatch & Schroeder, 2014). While none of the interventions in our sample explicitly focused on increasing the proportion of women teachers in hard-to-staff schools, several studies provide insight both on the challenge and on the impact of incentives as a solution.

Studies on teacher preferences highlight the potential and the limitations of financial incentives and other similar packages to attract women teachers to remote areas. A discrete choice experiment in India found that teachers who are women would require a salary differential of between 24 and 73 percent to move to a remote village, depending on whether they were originally from a rural or urban location (Fagernäs & Pelkonen, 2012). This is higher than the size of most financial incentives offered by real-life programs in our sample (Table 5). On the other hand, another discrete choice experiment, this time in Ghana, reported that women teachers preferred other incentives such as study leave with pay, expedited promotion and provision of housing over rural incentive allowance (Gad, 2015). Finally, a qualitative study with women teachers in Kenya reported support for both provision of housing and hardship allowance as viable strategies to attract teachers to remote areas (Kamere, Makatiani & Nzau, 2019).

Three of the fifteen studies in our sample provide gender-disaggregated impacts. Despite the potential preferences reported above, none of these programs were effective at recruiting women teachers. The rural hardship allowance in the Gambia did not increase the share of women teachers in target schools, but it reported positive teacher recruitment outcomes in general, suggesting that it increased recruitment for women and men teachers roughly equally (Pugatch & Schroeder, 2014). The behavioral intervention in Peru reported improved teacher recruitment on average, but the positive effects were driven by men (Ajzenman et al., 2021a). Finally, the teacher recruitment drive in India encouraged the appointment of women teachers—so that each school would have one man and one woman teacher—but the share of women teachers in target schools did not increase (Chin, 2005). These findings suggest that while incentives can be effective in some settings and for some women teachers, more work is needed to identify effective ways to improve teacher gender ratios in disadvantaged schools.

### 5.5 Findings from non-experimental studies

Our search unearthed twelve studies that were excluded from our final sample of experimental and quasi-experimental studies for methodological reasons—e.g., because they lacked a control group or simply reported on a survey of preferences—but which provide insight into programs aimed at staffing hard-to-staff schools in a wider range of countries (Table A.4). In Ghana, Cobbold (2006) describes a scheme in which rural districts sponsor candidates from their districts for teacher training and then contract them for three years, the idea being that candidates from rural districts might be more likely to remain in postings close to their homes. However, interviews with 12 teachers found that while they all appreciated the program, none of them planned to remain in the districts after their initial contract. In rural South Africa (North West province), most teachers report that the financial allowance is a motivating factor for them, both to work in their current schools and to show up to class (Poti, Mutsvangwa & Hove, 2014).

In China, Zhai et al. (2019) characterize a program that provides both “carrots” (free education and stipends) and “sticks” (a ten-year contract). Among teacher trainees, they find survey evidence that the policy boosts willingness to work in hard-to-staff schools. In 2014, China launched a policy that sent eligible urban teachers to short-staffed rural schools for a fixed period of time and compensated the participating teachers with a transportation subsidy, professional awards, and early promotion (Liao et al., 2019). However, school principals in rural areas reported that the relocated teachers were often tardy and refused to attend school meetings or teach on Fridays, and that the principals did not have the administrative authority to hold them accountable. Another two studies report on a program, also in China, that recruits graduates from elite universities to work in disadvantaged schools and finds that those who do so are more likely to participate because of altruistic reasons (Yin et al., 2019; Yin & Mu, 2020). Finally, a program that directly recruits college students to teach in rural schools faced challenges by teachers such as insufficient support from community, heavy workload, and low teacher pay (Li and Xue, 2021).

Some studies asked teachers or teacher trainees to rank possible incentives. Surveys with teacher trainees in Lao PDR and Cambodia reveal a high valuation of amenities, such that—for example—teachers would require between a 10 and 11 percent salary increase to compensate for each additional hour travel time from the nearest town. Among other amenities, a lack of electricity required the highest hypothetical compensation: 73 percent higher salary in Cambodia and 158 percent in Lao PDR (Sisouphanthong, Suruga & Kyophilavong, 2020). In India, teachers originally from urban areas are more averse to moving to rural locations than those from rural areas (Fagernäs & Pelkonen, 2012). In Ghana, teacher trainees stated that they preferred alternative benefits such as housing, expedited promotion, or study leave with pay over a hardship allowance (Gad, 2015). A qualitative study of women teachers in Kenya found support for both hardship allowance and free housing (Kamere et al., 2019). One of the studies included in our main sample, Swai (2013), also includes evidence from a small survey in which teachers rate various program components—cash incentives, housing, and other in-kind incentives (like bicycles or beds)—as equally important in drawing them to their rural postings.

Lastly, a survey in Malawi reveals one of the greatest weaknesses of these surveys. Rural teachers were split on whether they believed the hardship allowance they received led them to remain at their current school. Urban teachers were much more supportive of housing provision as an incentive than rural teachers, perhaps—as the authors posit—because rural teachers were acquainted with the actual poor quality of the housing provided (Mwenda & Mgomezulu, 2018). This signals the limitation of teachers or teacher trainees rating hypotheticals about options when they lack concrete knowledge.

### 5.6 Costs and cost-effectiveness

Most of the 15 included studies report on financial incentives in the form of bonuses or salaries. Teacher compensation already tends to make up a high percentage of education budgets in low- and middle-income countries, so understanding the cost implications is particularly important. We summarize the available cost data in Table 5.

**Table 5:** Available Cost Data on Programs to Staff Hard-to-Staff Schools.

Study	Country	Cost analysis
Bobba et al. (2022)	Peru	Teachers in extremely rural schools receive up to S/500 a month which is equivalent to 30 percent of contract teachers’ salary and between 20 and 30 percent of permanent teachers’ salary. In addition, they present cost-efficiency frontier graphs for filling every vacancy (no quality restriction) and filling every vacancy with teachers of equivalent competence as the average urban teacher.
Cabrera and Webbink (2020)	Uruguay	Bonus for teachers in schools in poor communities is up to 26% of base salaries.
Camelo and Ponczek (2021)*	Brazil	"a sizeable wage premium (24% to 36%)" for teachers in disadvantaged schools
Castro and Esposito (2022)	Peru	Teachers in extremely rural areas received about USD 70 every month in 2014, raised to USD 176 every month in 2015. Teachers in less rural but still eligible areas received between USD 25 and USD 35 every month for both years. The difference of USD 141 between the extremely rural area and the least rural area represents a 26% increase of a teacher's starting salary.
Chelwa et al. (2019)	Zambia	"rural hardship allowance corresponding to 20% of the base salary"
Elacqua et al. (2022) *	Chile	Teachers in disadvantaged schools received an additional 40% of the competency-based award value (equivalent to 16 percent of a teacher's average annual salary)
Hinze-Pifer and Méndez (2016)*	Chile	Teachers in disadvantaged schools receive between 4 percent to 30 percent over their base salary depending on the “difficulty score” of the school.
Pugatch and Schroeder (2014, 2018)	Gambia	Teachers who choose to teach in remote schools receive between 30 and 40 percent salary bonus. "At a cost of approximately US$350,000 annually, these additional teachers cost US$2500 each to recruit."
Rosa (2019)*	Brazil	São Paulo city pays "wage-premiums [5%-7%] for teachers working in schools in selected neighborhoods" farther from downtown.
Swai (2013)	Tanzania	cash incentive including signing bonus of between USD 179 to USD 357 (equivalent to at least one month take home pay), accommodations, and other inputs
Urquiola and Vegas (2005)	Bolivia	salary bonus of about 12. 5 percent of total compensation available (for comparison, the base salary of the paid teacher - interim teacher in an urban area - is USD 65 in 2002 or about 40 percent of total wage bill). Additional bonus is available for teachers depending on seniority and trainings received.
*Other intervention*s		
Ajzenman et al. (2021a)	Peru	“The cost of filling a teaching vacancy in a disadvantaged school using either [of the two information interventions] is approximately USD 13 per vacancy.” The existing national reward system highlighted by the information intervention can go up to twice the lowest salary level (which is about USD 650).
Ajzenman et al. (2021b)	Ecuador	$0 (zero-cost intervention)
Chin (2005))	India	The teacher component of the Operation Blackboard cost “cost $300 million from 1987 to 94 in 1994 U.S. dollars)” to recruit 140,000 teachers. “The central government pays the salary of the second teachers only for the initial few years. The state governments must pay the salary for subsequent years.”

Notes: *Although Elacqua et al. (2022) and Hinze-Pifer and Méndez (2016) both evaluate bonus for schools with disadvantaged status in Chile, they contextualize the incentive programs differently. Elacqua et al. (2022) frame the disadvantage school bonus as an add-on to the competency-based award available for all teachers regardless of location while Hinze-Pifer and Méndez (2016) cite up to thirteen bonuses (which includes both the disadvantage bonus and the competency bonus) on top of the basic renumeration for teachers. For the two incentive programs in Brazil, Rosa (2019) evaluates an incentive bonus established in the 1990s for São Paulo, Brazil while Camelo and Ponczek (2021) evaluate a more widely available incentive bonus launched in 2018 but limits the analysis of impact to schools in São Paulo due to challenges in data availability.

Teacher incentives tend to be reported either as a percentage of increase over base salary—such as the rural hardship allowance in Zambia that corresponds to 20 percent of the teachers’ base salary (Chelwa, Pellicer & Maboshe, 2019)—or in absolute amounts such as the USD 70 monthly bonus received by teachers in extremely rural schools in Peru in 2014 (Castro & Esposito, 2022). One study in Tanzania reports a one-time signing bonus equivalent to at least one month of take-home pay (about USD 179 to USD 357) (Swai, 2013).

The amounts reported vary dramatically. In Brazil, the disadvantage premium implemented in São Paulo ranges from 5 to 7 percent of teachers’ initial wages (Rosa, 2019) which is significantly smaller than other incentive programs, primarily because the wage premium was established in 1991 (about 30 to 50 percent of teachers wages at that time) and has not been amended or corrected for inflation at the time of the evaluation 20 years later. At the upper end of the range, the most competent teachers in Chile received an award of 33 percent of their base salary, plus an additional 40 percent of that award if the teachers chose to go to a disadvantaged school (Elacqua et al., 2022). Finally, the rural hardship allowance in the Gambia provided a salary bonus of 30 to 40 percent, yielding a cost of USD2,500 for recruiting one additional teacher, based on the total annual cost of the program and the number of qualified teachers recruited in hardship schools (Pugatch & Schroeder, 2018; 2014).

Bobba et al. (2022) evaluate a program that provides a rural bonus of up to 30 percent of teachers’ salary in Peru. They also model the cost-efficiency of filling every vacancy with either regular teachers (no restriction on qualification) or teachers of equivalent competence as the average urban teacher (i.e., a quality upgrade for rural schools). Unsurprisingly, they find that attracting competent teachers to rural schools would cost significantly more than just filling up all vacancies. It would take about twice the total national current budget for teachers’ wages to fill almost every vacancy (with only about half of the vacancies filled with a competent teacher), while it would take up to six times the total wage budget to close the teacher quality gap between rural and non-rural schools. In addition, they model an alternative policy that increases the supply of local teachers in the most disadvantaged locations (an increase of about 3 percent relative to the whole teacher applicant pool), taking into account revealed teacher preferences, including the willingness of teachers from rural areas and ethnic minorities to work in communities with similar profiles, and find that it could potentially save between 30 and 35 percent of the projected wage bill (i.e., a reduced but still massive sum) to completely close the teacher gap.

The recruitment drive in India for additional teachers cost the government USD 300 million (in 1994 dollars) between 1987 and 1994 to recruit 140,000 teachers which translates to USD 2140 per teacher (Chin 2005), not too different from the USD 2500 price tag for recruiting a rural teacher in the Gambia (Pugatch & Schroeder 2018; 2014).

Unsurprisingly, the behavioral interventions are much cheaper. The intervention in Ecuador was labeled as zero-cost since the treatment only requires re-ordering of schools in the job application portal which would require a one-time programming effort with no significant increased cost for rolling-out nationwide (Ajzenman et al. 2021b). The two alternative information interventions in Peru both cost approximately USD 13 to fill one teacher vacancy (Ajzenman et al. 2021a). If we look at outcomes that are comparable between these extremely cheap behavioral interventions and teacher financial incentives, we find that while behavioral interventions are not likely to close the quantity or quality gap between hard-to-staff schools and other schools, they are likely a cost-effective step in the process.

## 6 Discussion

### 6.1 Common challenges across these programs

In this section, we report implementation challenges cited by the 15 eligible studies. The behavioral interventions included in our review are more straightforward and would likely not require significant sustained investment over time. In contrast, financial incentives have multiple moving parts and often rely on—and are therefore limited by—existing civil service infrastructure.

#### 6.1.1 Timing and reliability of receiving payments

Any financial incentive program is subject to the limitations of the administrative system that houses it. Incentives to work in hard-to-staff schools are no exception. Teachers in Tanzania report delays in receiving the promised bonuses and a lack of facility to address concerns and follow-up on issues related to bonuses (Swai 2013). In one province of South Africa, teachers also report long delays in rural bonus payments and an unwanted yearly disbursement schedule instead of a preferred monthly payment (Poti et al. 2014). In Ghana, teachers report delays in payment, discrepancy between what was promised and what was paid out, and piecemeal payments instead of lumpsum payment (Cobbold 2006). In Zambia, delays in salary payment are also associated with teachers departing for better schools (Chelwa et al. 2019). Addressing bonus payment delays can be challenging if they stem from more systemic flaws such as generally late salary payments for civil service workers or lack of a central office to receive and process payment issues, but some of these concerns—such as the expected frequency of payments—could be a communication gap that could be corrected at lower costs.

An analogous challenge may be exacerbated with non-financial benefits, although those are not explicitly evaluated in our included studies. In Uruguay, non-salary benefits such as “additional time for coordination between teachers” and training sessions were supposedly a part of the program along with the financial incentive, but the non-salary components were not strictly enforced (Cabrera & Webbink 2020).^11^

#### 6.1.2 Information

In some cases, teachers may simply lack information about the incentives in place to encourage them to work in rural schools. Focus groups with teachers in Peru revealed that teachers were aware of the financial incentives, but they did not know which schools would qualify (Ajzenman et al. 2021a). In São Paulo, Brazil, information about wage premia at hard-to-staff schools was not included in official hiring documents, such that teachers may again have been unaware of which schools benefited from the program (Rosa 2019). In one Chile study, the authors posit that teachers may not be aware of the wage premium in disadvantaged schools until they begin teaching there, which may explain the positive impact of the wage premium on retaining teachers but not on attracting teachers (Elacqua et al. 2022).

In one intervention in Peru, the web-based application in which teachers identified which schools they would be willing to work in included simple icons like a money bag to indicate the presence of monetary incentives or a ladder to indicate the promise of faster career progression (Ajzenman et al. 2021a). Simple informational interventions like that may solve some information challenges.

#### 6.1.3 Other logistical implementation challenges for financial incentives

Aside from the timing of and information about payments, other logistical challenges in designing and implementing components of the financial incentive programs may blunt potential positive effects on recruitment and retention. The incentive program in São Paulo, Brazil—evaluated in 2010—was hampered by outdated eligibility designations (assigned in 1991), and an outdated bonus value (1990s wage rate), which potentially led to indifferent teacher recruitment and retention response (Rosa 2019).

Many of the interventions use eligibility criteria that are straightforward to measure, communicate, and validate, such as municipal-level socioeconomic scores, GPS-computed distance from roads, or population counts, but these may still miss capturing relevant aspects of hardship. Countries may include incentives for some types of hardship (rural schools) but not for other types (high-poverty urban schools). In Zambia, the GPS-computed distance from city centers failed to reflect natural barriers such as mountain ranges which increased actual travel distance to the school (Chelwa, Pellicer & Maboshe 2019). The program did allow for an appeal process and subsequent re-classification of schools. The same evaluation also reported inaccuracies in payroll data—teachers were still listed in schools where they no longer taught—which complicated the validation of compliance and subsequent evaluation of impact. Similar to cash transfers and other benefit programs, designing eligibility thresholds for teacher incentives in hard-to-staff schools in contexts with limited or unreliable administrative data will mean contending with the trade-off between errors of exclusion (schools with significant hardship that do not pass the eligibility cut-off) and errors of inclusion (non-hardship schools that qualify and receive incentives) and limited resources for monitoring compliance.

The duration for which schools are guaranteed hardship status can also influence how teachers rate the incentives’ attractiveness. In one program in Chile, for example, schools had to apply and be approved for hardship status every two years (Hinze-Pifer & Méndez 2016). On the one hand, this ensures that the distribution of hardship status is accurate and current, as opposed to outdated eligibility assignments reported in other programs. However, this can introduce a new information challenge, since in the absence of explicit action, teachers may only become aware of the change of status when they stop receiving the bonus, as happened in another program in Chile (Elacqua et al. 2022). In addition, short-lived incentives with high administrative burdens might affect the attractiveness of the bonus both for recruiting new hires but especially for retention, as has been documented for teacher incentives in high-income countries (See et al., 2020; See, Morris, Gorard, Kokotsaki, & Abdi, 2020) and for health workers in rural areas in low- and middle-income countries (World Health Organization 2020), where incentives are only effective in retaining workers as long as the incentives last.

Finally, systems have to decide which teachers qualify for the benefits associated with working at hard-to-staff schools. In Peru, many candidates who take advantage of the incentives to work in rural schools are temporary teachers who have not yet passed the national teacher qualification test (Bertoni et al. 2021). This may propagate inequalities across schools by drawing less qualified teachers to disadvantaged schools. Across the incentive programs evaluated in our sample, almost half report that both permanent and temporary contract teachers are eligible.^12^

### 6.2 Findings in relation to evidence from high-income countries

Staffing challenges in more remote or otherwise disadvantaged locations are not unique to low and middle income countries, and there are some common themes from the literature in high-income countries. In a recent systematic review, See et al. (2020) find 20 quantitative evaluations of interventions to improve retention on hard-to-staff schools; all of those with clear causal inference are from the United States.^13^ As we find in low- and middle-income countries, See et al. (2020) find a paucity of evidence on levers beyond financial incentives: “There is no good evidence yet that other approaches such as mentoring and induction, teacher development and alternative routes into teaching work for recruitment and retention, in high-need areas.” There is much to learn—across countries of all income levels—about the impact of different policy levers on recruitment and retention of teachers in hard-to-staff schools.

On financial incentives, the evidence that See et al. (2020) identify suggests that financial incentives are effective at boosting teacher recruitment. On retention, however, the authors found no lasting effect beyond the stipulated duration of the funding in most studies. The financial incentives in the experimental and quasi-experimental studies in our review are recurring, and the studies are not long term enough to speak directly to this. However, this finding is consistent with the qualitative work in Ghana that suggests that teachers would leave rural posts once their contracts were complete (Cobbold 2006).

While the broad pattern of results is similar between high-income countries and low- and middle-income countries, there are key differences to consider when comparing studies across settings. First, the average level of teaching complements (like books and blackboards) and other amenities (like access to health services) for teachers in remote schools in low- and middle-income countries is lower than that in high-income countries. Second, the gradient in these amenities between rural schools and urban schools may be different across the two settings, with many rural settings in high-income countries experiencing less of a scarcity of amenities relative to urban areas than in low-income countries. Third, monitoring and accountability systems in high-income countries may make basic elements of teacher performance, like day-to-day absenteeism, less of a challenge.

All of these elements fall on a continuum across the income level of the setting and other factors, such as public and private investments in education. As a result, drawing lessons from similar settings may be more relevant than a binary distribution into high-income country systems and low- and middle-income country systems. We focus on the latter experiences in this review for simplicity, but some upper middle-income countries may draw important lessons from the evidence on staffing challenging schools in high-income countries.

### 6.3 Methodological considerations

Ten of the studies considered in this review use regression discontinuity strategies (Table 3), and as discussed in Section 5.2.1, regression discontinuity strategies may overstate the impact of efforts to staff schools if teachers on the treated side of the discontinuity are drawn from the untreated side of the discontinuity. Taking financial incentives as an example, one solution—following Castro and Esposito (2022)—is to use variation in the proximity of schools that are not eligible for the financial incentive to schools that are eligible for the financial incentive to identify spillover effects. Eligible schools that are close to ineligible schools are more likely to draw teachers from the ineligible schools than eligible schools that are far away. These spillovers pose a challenge to calculating the local average treatment effect.

Beyond that challenge, providing incentives (whether financial or other) for teachers to move to hard-to-staff schools has implications for the whole education system (i.e., the average treatment effect). Without an increase in teachers overall, more teachers in remote schools means fewer teachers in urban schools, and while this might be equity enhancing, it may not indicate an improvement for all students in the system (i.e., a Pareto improvement). Looking beyond education, incentives that draw more professionals into the teaching profession may or may not increase social welfare for the society as a whole, since those teachers may be drawn from contributing to other aspects of the economy, with either higher or lower social returns.^14^ Understanding how incentives affect not just the distribution of teachers but also the overall supply of teachers—and from which areas of the economy they are drawn—is an area that merits further research in the future.

### 6.4 Limitations to this work

This work faces several limitations. First, the included studies are from Latin America (six countries), Africa (two countries), and South Asia (just one country). Staffing rural or high-poverty schools is a challenge around the world, as evidenced by descriptive work highlighted in Section 2, but we lack evidence of the effectiveness of schemes from the Middle East, other parts of Asia and elsewhere. Second, this review is limited by the interventions that have been studied. Countries have implemented a wide range of activities to attract or retain teachers in rural schools (Elacqua et al. 2018; McEwan 1999; Pugatch & Schroeder 2014), and teachers signal that various of these programs could potentially be effective (Gad 2015; Kamere, Makatiani & Nzau 2019; Swai 2013), but most of these policies remain unevaluated. Almost all the evidence is focused on financial incentives, with one evaluation of an earlier teacher recruitment drive and a couple of recent evaluations of behavioral strategies. Much remains unknown on the effectiveness of the wider range of policies. Future researchers can both document these policies in more detail and evaluate them. Third, this review focuses on interventions implemented through government systems. However, there is some evidence on improving either the quantity or quality of teachers from non-government interventions. We briefly discuss that evidence in Appendix Section A.4.

## 7 Conclusion

In this systematic review, we report on the results of 15 experimental or quasi-experimental evaluations of interventions to recruit or retain teachers in hard-to-staff schools in low- and middle-income countries. We find mostly positive impacts of financial incentives on teacher outcomes, and we find suggestive evidence of positive impacts on student learning and attendance. We report promising evidence from recent evaluations of behavioral interventions, providing information about existing incentives or increasing the salience of hard-to-staff school options. Recruiting teachers who are women to hard-to-staff school poses a particular challenge. We also provide new evidence that teachers in rural areas in many countries tend to be less skilled and more often absent than their urban-based counterparts.

Future work in this area may proceed along at least two lines. First, governments draw on a wide array of policies to attract or retain teachers in hard-to-staff schools, but almost all of the evaluations of large-scale government policies have been of financial incentives. Research evaluating the impact of alternatives—both informational and behavioral interventions, as reported in this review, and alternative policies such as speedier promotion or subsidized, secure housing—will expand the evidence-based toolbox for policymakers seeking to support teachers in reaching the most disadvantaged students. Second, most of the studies do not account for spill-over or general equilibrium effects. Research exploring these effects can help ensure these programs are not just re-allocating teachers from schools that are slightly less disadvantaged to those that are slightly more disadvantaged.

Even with the benefit of low-cost behavioral interventions, fully staffing hard-to-staff schools with effective teachers is unlikely to be cheap. Current levels of financial incentives are insufficient to close gaps completely. But with most out-of-school children currently residing in rural areas and children in high-poverty schools achieving the lowest test scores, education systems will need to draw on an array of strategies to strengthen their overall educational performance and invest in broad-based human capital.

## Data Availability

No data was used for the research described in the article.

## Appendix Supplementary materials

Image, application 1

## Footnotes

1 A related literature explores the recruitment and retention of doctors and nurses in challenging posts, where many countries have implemented similar policies (Araújo and Maeda 2013; World Health Organization 2020).

2 See Appendix Section A.1 for a discussion of the data for this analysis.

3 Appendix Section A.3 provides detail on how we identified articles to characterize the problem.

4 McEwan (1999) separates these into monetary inducements—a compensating wage differential—or into benefits that make rural teaching jobs “less rural,” such as improved teacher housing or better teacher professional development opportunities.

5 Pugatch and Schroeder (2014) allude to a list of policies in footnote 3 of their published paper, available upon request. That is the list we drew from.

6 Discrete choice experiments are a research method that asks survey respondents to choose between alternative hypothetical scenarios in order to better understand preferences (Mangham, Hanson, and McPake 2009). In the context of staffing hard-to-staff schools, a discrete choice experiment might ask a teacher training student to choose between a certain wage in an urban setting and a higher wage in a remote setting. By testing different wages, one can understand candidates’ stated preferences. These may not map perfectly to their preferences when faced with real life scenarios.

7 Not all researchers would characterize matching studies as “quasi-experimental” because the assumptions required to establish causality are stronger. However, we do include them here because matching studies seek to emulate experimental designs, albeit with highly varying degrees of credibility.

8 In addition to recruiting and retaining effective teachers in hard-to-staff schools, another approach is to seek to boost the performance of teachers already in hard-to-staff schools, as in Gaduh et al. (2021). A full review of such complementary interventions is beyond the scope of this review.

9 The one study that does not report teacher-related outcomes (Pugatch and Schroeder 2018) is complemented by another study by the same authors, using the same experiment, which does report teacher-related outcomes (Pugatch and Schroeder 2014). So to be precise, all interventions report teacher outcomes, even though not all studies do.

10 This counterintuitive finding is consistent with the strong evidence on the importance of teacher quality (Araujo et al. 2016; Buhl-Wiggers et al. 2019) and the weaker, more mixed evidence on the impact of pupil-teacher ratios (Banerjee et al. 2007; Duflo, Dupas, and Kremer 2015). However, another possible explanation may have more to do with student rather than teacher movement: lower-performing students may have dropped-out of control schools in that timeframe.

11 These other, non-salary incentives may be at least as difficult to implement effectively as financial incentives, as evidenced by the experience of many countries with teacher professional development (Popova et al. 2022).

12 Specifically, six report that incentives are available for teachers on permanent and temporary contracts (Bobba et al. 2021; Camelo and Ponczek 2021; Castro and Esposito 2022; Elacqua et al. 2022; Hinze-Pifer and Méndez 2016; Urquiola and Vegas 2005). Three report that incentives are only available for teachers on permanent contracts (Ajzenman et al. 2021a; Ajzenman et al. 2021b; Swai 2013). The others do not report this information.

13 The one non-U.S. study is from Brazil, but it evaluates a reform that coincides with other significant educational reforms and so isolating the causal impact is challenging.

14 This argument is discussed in detail in Section 5 of De Ree et al. (2018).
