# Supplementary material for: How to recruit teachers for hard-to-staff schools: A systematic review of evidence from low- and middle-income countries
Source: Econ Educ Rev. 2023 Aug;95:None. doi: 10.1016/j.econedurev.2023.102430 (PMC10398677; doi:10.1016/j.econedurev.2023.102430)
Supplement: Supplementary file 1 [file mmc1.pdf]

Supplementary Appendix for

"How to Recruit Teachers for Hard-to-Staff Schools: A Systematic Review of Evidence from Low- and Middle-Income Countries"

by Evans and Mendez Acosta

This appendix includes the following materials:

- Appendix Section A.1: Additional information on Service Delivery Indicators data and analysis
- Appendix Section A.2: Country contexts
- Appendix Section A.3: Methods to characterize the problem
- Appendix Section A.4: Non-government interventions
- Appendix Section A.5: Additional tables and figures
- Appendix Section A.6: Appendix references

Appendix Section A.1: Additional information on Service Delivery Indicators data and analysis

The World Bank's Service Delivery Indicators datasets use a multistage cluster sampling design across rural and urban areas and public and non-public schools, and they report a wide range of information on teachers' workload and performance along with school-level variables. For this study, we looked at differences in teacher absenteeism and teacher skills between rural and urban areas. Absenteeism rates are calculated from the attendance records of an unannounced visit of an enumerator to schools (Service Delivery Indicators 2017). The SDI surveys also evaluate the teachers' minimum knowledge required to be effective in reading, writing, arithmetic, and pedagogy by administering task-based tests to all mathematics and language teachers who taught fourth grade in the year the survey was conducted or third grade in the previous year (Service Delivery Indicators 2017). The test covers the literacy and numeracy curriculum expected to be taught at the lower primary level. We used the observed classroom size data available to calculate student-teacher ratios. Finally, we used the data on students with at least one exercise book and classrooms with functioning boards (i.e., blackboards with enough contrast for students at the front and back of the classroom to read what is written on them) to analyze available classroom resources. Averages were computed at the school-level and aggregated at the country level using school-level weights. We compare these indicators for schools in rural areas versus schools in the same country located in urban or semi-urban areas.

Appendix Section A.2. Country contexts

Challenges in staffing are almost universal, but countries face different specific challenges based on their contexts. For example, among the eight countries for which we have Service Delivery Indicators, the share of schools in rural areas are uniformly high but there is considerable range (Figure A.4). The discrete classification of rural versus urban (often based on population count) may mask some of the dynamics in accessing services and challenges in existing transportation infrastructure. In Tanzania, where 83 percent of schools are in rural areas, almost 40 percent of the population either live in cities and towns or are able to access these centers within an hour of travel (Cattaneo et al. 2021). Across the 17 countries where our quasi-experimental and descriptive studies of interventions have been implemented, we see a wide range of variation in access (Figure A.5).

More than 70 percent of the population in Bolivia, Brazil, Ecuador, Gambia, Ghana, Peru and Uruguay live in cities, towns or areas within an hour of travel from these cities and towns, but less than half of the population do so in Kenya. In Laos, only 16 percent live in or near these centers while almost a quarter of the population live more than three hours from any of these urban centers. Effective interventions to attract and retain teachers might look different across these contexts and most likely would demand different implementation strategies and price tags.

Appendix Section A.3: Methods to characterize the problem

Beyond summarizing the impacts of interventions, we also characterize the problem of staffing hard-to-staff schools. We do this in two ways. First, we draw on the studies identified in our systematic search (detailed in Section 4 of the article) and summarize how they characterize the problem across a range of countries.

Second, while this study focuses on staffing hard-to-staff schools across all low- and middle-income countries, our original analysis of data to help quantify the problem focuses on African countries because of newly available data in those settings. The Service Delivery Indicators (SDI) program collects and reports nationally representative cross-sectional surveys on service delivery performance in education and health facilities across select countries in Africa (Bold et al. 2011; 2010). Currently, there are nine publicly available education datasets for eight countries (World Bank Microdata Library 2021): Kenya (2012), Madagascar (2016), Morocco (2016), Mozambique (2014), Nigeria (2013), Tanzania (2014 and 2016), Togo (2013) and Uganda (2013). We use these surveys to examine teacher skills and teacher absence in urban and rural areas, as described in Appendix Section A.1.

Appendix Section A.4: Non-government interventions

This paper focuses on interventions implemented by the government in recruiting teachers to disadvantaged schools, but several evaluated interventions to address the teacher gap have been implemented by non-state actors, often in public schools.

One such class of interventions involves recruiting teachers who are not part of the civil service directly from communities and for a fixed period of time. These teachers are often referred to as "contract teachers." While contract teachers have been employed widely by governments (UNESCO 2020), they have largely been evaluated in the context of non-government interventions. These teachers may either be individuals with formal teacher training but no civil service position—as in a study in Kenya (Duflo et al. 2015)—or community members who receive basic training before assisting with classes—as in a study in India (Banerjee et al. 2007). Both the Kenya and India interventions had positive impacts on student learning, particularly for students taught by the contract teachers. This may be in part because teachers on temporary contracts may be at greater risk of losing future contracts due to nonperformance. In Kenya, however, the government subsequently implemented a contract teacher intervention, and the impacts on student learning were indistinguishable from zero (Bold et al. 2018). A large-scale contract teacher intervention implemented by a non-government organization in the same study delivered positive impacts, suggesting that the difference is more likely to be administration than scale. In the government program, the national teachers' union sued for civil service positions for the contract teachers, such that the incentive to perform in order to achieve contract renewal may have been dampened.

Another set of interventions includes programs that aim to provide potentially high performing candidates with an expedited path into teaching. One prominent example, the Teach for All initiative, recruits high-performing university graduates from non-teaching backgrounds, provides them with pre-service training and on-going mentorship, and deploys them to disadvantaged schools for a pre-determined period, usually two years (Cumsille and Fiszbein 2015; Teach for All 2021). A preliminary evaluation of Chile's version of the program reported positive effects on student test scores as well as students' self-esteem, self-efficacy, and other non-cognitive skills (Alfonso et al. 2010). An evaluation in Peru reported mostly positive test score impacts (Lavado and Guzmán 2020), and an evaluation in Mexico showed that the program

reduced student tardiness and absenteeism and improved students' socio-emotional skills (Chacón and Peña 2017).

Appendix Section A.5: Additional tables and figures

Table A.1

Teacher Language Competency Score in Urban and Semi-Urban vs. Rural Areas in Selected Low- and Middle-Income Countries

Score in language task (out of 100%)	Urban/ Semi-urban	Rural	Difference (rural - urban/semi-urban)	Robust SE	Statistical significance
Kenya 2012	63.9%	63.2%	-0.66%	1.20%	
Madagascar 2016	26.0%	19.7%	-6.32%	1.09%	***
Morocco 2016	39.9%	40.0%	0.10%	1.13%	
Mozambique 2014	31.9%	30.8%	-1.14%	2.12%	
Nigeria 2013	34.5%	27.6%	-6.88%	1.28%	***
Tanzania 2014	37.1%	33.4%	-3.66%	1.46%	**
Tanzania 2016	29.8%	27.3%	-2.45%	0.87%	***
Togo 2013	51.8%	47.9%	-3.84%	2.05%	*
Uganda 2013	56.6%	53.0%	-3.54%	0.01%	**

Source: Authors' analysis using data from the World Bank's Service Delivery Indicators Education Survey.
Note: The teacher numeracy competency score is the percent answered correctly in a task-based assessment based on lower-primary level curriculum (scores are out of 100 percent). Averages are first computed at the school-level and aggregated at the country level using school-level weights. Differences in rural and urban/semi-urban rates are tested using bivariate regressions with school-level weights and robust standard errors and are presented here with statistical significance: * significant at 10%; ** significant at 5%; *** significant at 1%.

Table A.2

Teacher Numeracy Competency Score in Urban and Semi-Urban vs. Rural Areas in Selected Low- and Middle-Income Countries

Score in math task (out of 100%)	Urban/ Semi-urban	Rural	Difference (rural - urban/semi-urban)	Robust SE	Statistical significance
Kenya 2012	75.5%	78.4%	2.93%	1.97%	
Madagascar 2016	23.2%	24.1%	0.93%	1.26%	
Morocco 2016	51.5%	67.5%	16.00%	3.03%	***
Mozambique 2014	24.4%	27.5%	3.13%	2.21%	
Nigeria 2013	44.6%	35.0%	-9.60%	1.85%	***
Tanzania 2014	59.8%	59.5%	-0.32%	1.97%	
Tanzania 2016	61.8%	61.4%	-0.33%	1.67%	
Togo 2013	40.2%	28.8%	-11.44%	3.51%	***
Uganda 2013	61.0%	56.0%	-5.02%	1.73%	***

Source: Authors' analysis using data from the World Bank's Service Delivery Indicators Education Survey.

Note: The teacher numeracy competency score is the percent answered correctly in a task-based assessment based on lower-primary level curriculum (scores are out of 100 percent). Averages are first computed at the school-level and aggregated at the country level using school-level weights. Differences in rural and urban/semi-urban rates are tested using bivariate regressions with school-level weights and robust standard errors and are presented here with statistical significance: * significant at 10%; ** significant at 5%; *** significant at 1%.

Table A.3

Teacher Competency Score in Urban and Semi-Urban vs. Rural Areas in Selected Low- and Middle-Income Countries

Average score in pedagogy tasks (out of 100%)	Urban/ Semi-urban	Rural	Difference (rural - urban/semi-urban)	Robust SE	Statistical significance
Kenya 2012	35.7%	36.2%	0.44%	1.83%	
Madagascar 2016	18.2%	15.6%	-2.60%	1.62%	
Morocco 2016	19.9%	24.5%	4.63%	2.05%	**
Mozambique 2014	10.5%	14.4%	3.87%	1.76%	**
Nigeria 2013	14.9%	9.0%	-5.89%	0.92%	***
Tanzania 2014	38.1%	34.8%	-3.31%	1.71%	*
Tanzania 2016	21.1%	19.3%	-1.78%	1.12%	
Togo 2013	19.1%	15.6%	-3.45%	1.51%	**
Uganda 2013	25.8%	22.0%	-3.75%	1.09%	***

Source: Authors' analysis using data from the World Bank's Service Delivery Indicators Education Survey.

Note: The teacher pedagogy competency score is the average of percent answered correctly in task-based (scores are out of 100 percent). Averages are first computed at the school-level and aggregated at the country level using school-level weights. Differences in rural and urban/semi-urban rates are tested using bivariate regressions with school-level weights and robust standard errors and are presented here with statistical significance: * significant at 10%; ** significant at 5%; *** significant at 1%.

Table A.4

List of 12 Discrete Choice Experiments or Excluded Studies of Interventions

Study	Type of program	Country	Year of intervention evaluated
Cobbold (2006)	Financial incentive<br>(district-sponsored allowance for teacher trainers in return for a three-year contract to teach in the district)	Ghana	Introduced in 2000/2001 academic year
Fagernäs and Pelkonen (2012)	Financial incentive<br>(salary increase for moving to a remote location)	India	The discrete choice experiment was conducted in 2010.
Gad (2015)	Financial and non-financial incentives<br>(incentive packages such as granting of study leave with pay, provision of housing and promotion after three years of work)	Ghana	The discrete choice experiment was conducted in 2015.
Kamere et al. (2019)	Financial incentive and an administrative policy<br>(decentralization of recruitment to the school level and a separate policy on hardship allowance and housing)	Kenya	The decentralization policy signed in 2001. Hardship allowance and housing policy has been in effect since at least 2006.
Li and Xue (2021)	Non-financial incentive<br>(recruitment of college students to teach in rural schools)	China	The program called “Special post plan for School Teachers in Rural Compulsory Education” was introduced in 2006 by the national government
Liao et al. (2019)	Financial and non-financial incentive<br>(teacher rotation policy that sends urban teachers to rural areas and compensates them with transportation subsidy, professional awards, and early promotion)	China	The policy was issued by China’s central government in 2014.
Mwenda and Mgomezulu (2018)	Financial incentive<br>(teacher rural allowance)	Malawi	Policy is already in effect by 2009.
Poti et al. (2014)	Financial incentive<br>(teacher rural allowance)	South Africa	Policy signed in 2008.
Sisouphanthong et al. (2020)	Financial incentive<br>(teacher rural allowance)	Cambodia, Laos	The discrete choice experiment was conducted in 2014 in Laos and in 2015 in Cambodia.

Yin et al. (2019)<br>Yin and Mu (2020)	Non-financial incentive<br>(recruiting teachers from elite universities to serve in rural schools for a fixed period of time)	China	The program called "Elite Graduates as Rural Teachers" was launched in 2008.
Zhai (2019)	Financial incentive<br>(government-contracted preservice teacher program that provides free education, stipends and guaranteed employment in exchange for teaching in rural areas for at least two years)	China	Policy launched in 2007.

Table A.5

Teacher Outcomes from Studies on Incentivizing Teachers in Hard-to-Staff Schools

Study	Country	Intervention	Research Design	Timeframe	Data/Sample	Outcomes	Specification	Coef.	SE
Ajzenman et al. (2021a)	Peru	Treatment 1: "altruistic identity" intervention to prime teachers' social-oriented motivations<br>Treatment 2: "extrinsic incentive" intervention targeted information campaign promoting the existing incentives for teachers in underprivileged schools	Randomized controlled trial.	The incentive system was established in 2013.<br><br>The experiment was implemented in 2019 with outcomes measured shortly after the treatment.	Control: 3,861 teachers<br><br>Altruistic identity: 3,852 teachers<br><br>Extrinsic incentive: 3,855 teachers	Both treatments increased rate of teachers applying to disadvantaged schools (driven by male teachers; altruistic treatment has higher impact than extrinsic treatment). The altruistic treatment increased likelihood of actual assignment to disadvantaged schools (higher-performing candidates put disadvantaged	Treatment: Altruistic identity		
							Outcome: proportion of disadvantaged schools included in the teachers' choice set	0.019 **	0.0086
							Outcome: proportion of disadvantaged schools included in the teachers' choice set (male teachers only)	0.0346 **	0.0141
							Outcome: proportion of disadvantaged schools included in the teachers' choice set (female teachers only)	0.0107	0.0107
							Outcome: proportion of disadvantaged	0.0243 **	0.0122

	schools as primary choices) while the extrinsic treatment did not affect actual assignment (those who chose disadvantaged schools in this treatment were more likely to be lower-performing teachers and did not get their first choice).	schools included in the teachers’ choice set (baseline high performers only)		
		Outcome: proportion of disadvantaged schools included in the teachers’ choice set (baseline low performers only)	0.011	0.012
		Outcome: being an active teacher in a disadvantaged school in 2020	0.0198	0.0176
		Outcome: being an active teacher in a disadvantaged school in 2020 (male teachers only)	0.0444	0.027
		Outcome: being an active teacher in a disadvantaged school in 2020 (female teachers only)	0.0085	0.0245
		Outcome: being an active teacher in a disadvantaged school in 2020 (baseline high performers only)	0..0332	0.0232
		Outcome: being an active teacher in a disadvantaged school in 2020	0.0196	0.0258

(baseline low performers only)		
Treatment: Extrinsic incentive		
Outcome: proportion of disadvantaged schools included in the teachers' choice set	0.0201 **	0.0086
Outcome: proportion of disadvantaged schools included in the teachers' choice set (male teachers only)	0.0308 **	0.0138
Outcome: proportion of disadvantaged schools included in the teachers' choice set (female teachers only)	0.0154	0.0109
Outcome: proportion of disadvantaged schools included in the teachers' choice set (baseline high performers)	0.0132	0.0122
Outcome: proportion of disadvantaged schools included in the teachers' choice set (baseline low performers)	0.0249 **	0.012
Outcome: being an active teacher in a	0.0373 **	0.0165

							disadvantaged school in 2020		
							Outcome: being an active teacher in a disadvantaged school in 2020 (male teachers only)	0.057 **	0.0251
							Outcome: being an active teacher in a disadvantaged school in 2020 (female teachers only)	0.0267	0.0223
							Outcome: being an active teacher in a disadvantaged school in 2020 (baseline high performers only)	0.0569 **	0.0221
							Outcome: being an active teacher in a disadvantaged school in 2020 (baseline low performers only)	0.0338	0.0237
Ajzenman et al. (2021b)	Ecuador	Zero-cost nationwide government intervention that listed teaching vacancies in hard-to-staff schools were listed first on a job	Randomized controlled trial.	Program implemented in 2019 and evaluated immediately after the recruitment cycle.	18,133 teacher candidates (half to treatment and half to control)	Teachers were more likely to apply to disadvantaged schools, rank them as higher priority and be assigned to these schools. Teacher quality	Outcome: percentage of understaffed schools in choice set	0.013 **	0.006
							Outcome: share of understaffed school among first choice	0.052 ***	0.012
							Outcome: at least one understaffed	0.027 **	0.011

	application platform before other schools instead of alphabetically (no change in information or incentives).		did not affect response to treatment.	school among first 2 choices		
				Outcome: at least one understaffed school among first 3 choices	0.029 **	0.011
				Outcome: assigned to understaffed school	0.034 ***	0.012
				Outcome: accepted offer in understaffed school	0.031 **	0.012
				Outcome: percentage of understaffed schools in choice set (coefficient of interacting treatment and being low-performer)	-0.011	0.012
				Outcome: share of understaffed school among first choice (coefficient of interacting treatment and being low-performer)	-0.032	0.026
				Outcome: at least one understaffed school among first 2 choices (coefficient of interacting treatment and being low-performer)	-0.017	0.027

							Outcome: at least one understaffed school among first 3 choices (coefficient of interacting treatment and being low-performer)	-0.005	0.023
							Outcome: assigned to understaffed school	-0.024	0.024
							Outcome: accepted offer in understaffed school (coefficient of interacting treatment and being low-performer)	-0.0818	0.024
Bobba et al. (2022)	Peru	Government-sponsored wage bonus for teachers in select rural areas categorized by population and travel time to provincial capital computed by GPS. Bonus is up to S/500, equivalent to up to 30 percent of contract teachers' monthly earnings.	Regression discontinuity using population and distance cut-off.	Policy was first implemented in 2014 with increase in bonuses in 2015.<br><br>Study period is 2015-2018	The main results table report outcomes based on data from up to 925 schools.	Vacancies in bonus schools became more desirable for teacher applicants and the quality of recruited teachers improved but only for contract teaching positions.	Outcome: bonus school is in the list of teachers' preference list (permanent teaching position)	0.177 **	0.068
							Outcome: vacancy is filled (permanent teaching position)	-0.001	0.071
							Outcome: competency score of recruited teachers (permanent teaching position)	-0.014	0.175
							Outcome: rank of teacher that filled the vacant position (contract teaching position) (lower	-0.121 ***	0.035

							rank has higher competence)		
							Outcome: vacancy is filled (contract teaching position)	0.045	0.045
							Outcome: competency score of recruited teachers (contract teaching position)	0.451 ***	0.123
Cabrera and Webbink (2020)	Uruguay	Contexto Socio Cultural Crític (CSCC program) provides up to 26% increase in base salary for teachers working poor neighborhoods.	Regression discontinuity around the poverty index threshold.	The program was launched in 1995 and updated in 2005 to use a poverty index cut-off as eligibility criteria.<br><br>Outcome variables are available until 2013 with administrative data available since 1992 (study does not evaluate long-term effects but outcomes against program participation in	543 schools	Increased teacher tenure (length of staying in one school) by one year but no significant change in average teacher turn-over (teachers with more tenure are less likely to leave program schools than non-beneficiary schools). Increased average teacher experience by two or three years (teachers with more experience get recruited).	Outcome: average teacher experience	1.686 ***	0.304
							Outcome: average length of service of teachers	0.403 **	0.191

				year t-1 and t-2)					
Camelo and Ponczek (2021)	Brazil	ALE program (Adicional por Local de Exercício): "wage premium (24% to 36%) to teachers at disadvantaged schools" based on school location.	Regression discontinuity around the socio-economic index threshold.	Program was launched in 2008.<br><br>Data for analysis covers 2007 to 2012.	1,422 schools received compensation and 1,324 did not	Reduced teacher turn-over.	Outcome: teacher turnover (2008-09)	-0.048 *	0.026
							Outcome: teacher turnover (2009-10)	-0.064 **	0.028
							Outcome: teacher turnover (2010-11)	-0.028	0.030
							Outcome: teacher turnover (2011-12)	-0.083 **	0.034
							Outcome: teacher turnover (pooled)	-0.050 **	0.024
Castro and Esposito (2022)	Peru	Rural bonuses based on community population and distance from capital	Regression discontinuity along the school's distance in minutes to the nearest provincial capital (120 minutes) and number of inhabitants (500)	Started in 1990, revamped in 2014 to increase bonuses and include more schools.<br><br>Evaluated in 2016.	9,948 extremely rural schools (treatment group; receives the highest available bonus)<br><br>11,575 rural schools (control; receives smaller bonus)	The intervention reduced teacher attrition and reduced vacancies but with negative externality affecting control schools near treatment schools.	Outcome: teacher attrition (% of teachers that leave the school before the end of the academic year) (schools in communities with less than 500 inhabitants)	-1.460 **	0.865
							Outcome: teacher attrition in control schools less than 30 minutes away (schools in communities with less than 500 inhabitants)	1.351	0.966
							Outcome: teacher attrition (schools more than 120 minutes from road)	-4.846 ***	1.755

							Outcome: teacher attrition in control schools less than 30 minutes away (schools more than 120 minutes from capital)	1.350	1.483
							Outcome: teacher vacancies filled (schools in communities with less than 500 inhabitants)	1.569 *	0.935
							Outcome teacher vacancies filled in control schools less than 30 minutes away (schools in communities with less than 500 inhabitants)	-2.496 **	1.062
							Outcome: teacher vacancies (schools more than 120 minutes from road)	3.441 *	2.043
							Outcome: teacher vacancies filled in control schools less than 30 minutes away (schools more than 120 minutes from capital)	-3.147 *	1.767
Chelwa et al. (2019)	Zambia	Rural hardship allowance	Regression discontinuity	Allowance was implemented in	3,000 schools, about half	Some (but weak) positive effect on	Outcome: log teachers	0.045 *	0.026

		corresponding to a salary increase of 20% "allocated to schools outside a given radius from district centers."	by GPS-computed distance to district city centers.	the 1990s, revamped in 2008 (to become a 20% salary increase) and some implementation rules were changed in 2010.<br><br>Evaluation uses data from 2014 to 2015	received the allowance allocation; final sample is 137 schools (44 pairs across the threshold) due to challenges in implementation and data availability	teacher retention. Some (but weak) positive effect on stock of teachers.	Outcome: teacher tenure	0.29	0.218
							Outcome: share teachers transferred to other school	0.002	0.008
							Outcome: teacher education	0.11	0.016
							Outcome: teacher age	-0.054	0.224
Chin (2005)	India	Operation Blackboard is a government initiative to recruit additional teachers for one-teacher primary schools, complemented with provision of equipment packets (blackboards, books, maps, charts, toys, teacher's manuals, and other basic inputs) to all primary schools.	Difference-in-differences by birth cohort (children who started attending primary schools before and after 1987 when the program was launched) and state of residence (children in states with higher share of one-teacher schools were more exposed	Launched in 1987, the program served all originally targeted schools by 1994.	Recruitment drive for 140,000 teachers as second teachers to one-teacher primary schools	Only a quarter to a half of the teachers were sent to one-teacher schools; since average class size did not decrease, teachers are presumed to have shifted from larger schools to smaller schools instead of being newly recruited.	Outcome: proportion of primary schools with one teacher	-0.0498 ***	0.0122
							Outcome: proportion of primary schools with two teachers	0.0306 ***	0.0107
							Outcome: proportion of primary schools with three teachers	0.0075 ***	0.0021
							Outcome: proportion of primary schools with four teachers	0.0057 ***	0.0018
							Outcome: proportion of primary schools with five or more teachers	0.0053 **	0.0020
							Outcome: teachers per primary section (in primary school only - up to 5th grade only)	0.0869 ***	0.0247

			to the program)				Outcome: teachers per primary section (in primary sections excluding primary schools)	-0.6750 **	0.2928
							Outcome: teachers per primary section (in all primary sections)	0.0090	0.0529
							Outcome: pupils in grades 1 to 5 per primary section teacher)	1.1954	1.4076
							Outcome: population aged 6 to 10 per primary section teacher)	0.9065	1.3913
							Outcome: trained teachers as a percent of all primary section teachers	0.0016	0.0155
							Outcome: female teachers as a percent of all primary section teachers	0.0021	0.0044
Elacqua et al. (2022)	Chile	Additional incentive for teachers (additional 40% of the usual competency-based award) under the Pedagogical	Regression discontinuity - schools are considered disadvantaged if 60% or more of students are low income.	The version of AEP evaluated in the study has been implemented between 2012 and 2015. Evaluation	around 1,500 teachers across three years	Increased retention of talented teachers in disadvantaged schools but no impact on recruiting teachers who were not already	Outcome: probability of retention in the public system (t+1, all teachers, winning lowest award)	0.02	0.04
							Outcome: probability of retention in the	-0.02	0.03

	Excellence Assignment program (AEP)	uses data from 2011 to 2017.	working in disadvantaged schools at baseline.	public system (t+2, all teachers, winning lowest award)		
				Outcome: probability of retention in the public system (t+1, all teachers, winning middle award)	-0.01	0.03
				Outcome: probability of retention in the public system (t+2, all teachers, winning middle award)	0.02	0.03
				Outcome: probability of retention in the public system (t+1, teachers in disadvantaged schools at baseline, winning lowest award)	-0.06	0.06
				Outcome: probability of retention in the public system (t+2, teachers in disadvantaged schools at baseline, winning lowest award)	-0.03	0.05
				Outcome: probability of	-0.04	0.03

	retention in the public system (t+1, teachers in disadvantaged schools at baseline, winning middle award)		
	Outcome: probability of retention in the public system (t+2, teachers in disadvantaged schools at baseline, winning middle award)	0.06*	0.03
	Outcome: probability of retention in the public system (t+1, teachers in more advantaged schools at baseline, winning lowest award)	0.11*	0.06
	Outcome: probability of retention in the public system (t+2, teachers in more advantaged schools at baseline, winning lowest award)	0.00	0.02
	Outcome: probability of retention in the	0.03	0.04

		public system (t+1, teachers in more advantaged schools at baseline, winning middle award)		
		Outcome: probability of retention in the public system (t+2, teachers in more advantaged schools at baseline, winning middle award)	-0.02	0.06
		Outcome: probability of teaching in a high-achieving school (t+1, all teachers, winning lowest award)	0.20**	0.09
		Outcome: probability of teaching in a high-achieving school (t+2, all teachers, winning lowest award)	0.12	0.10
		Outcome: probability of teaching in a high-achieving school (t+1, all teachers, winning middle award)	0.15*	0.09

	Outcome: probability of teaching in a high-achieving school (t+2, all teachers, winning middle award)	0.12	0.09
	Outcome: probability of teaching in a high-achieving school (t+1, teachers in disadvantaged schools at baseline, winning lowest award)	0.07	0.15
	Outcome: probability of teaching in a high-achieving school (t+2, teachers in disadvantaged schools at baseline, winning lowest award)	0.03	0.13
	Outcome: probability of teaching in a high-achieving school (t+1, teachers in disadvantaged schools at baseline, winning middle award)	-0.15	0.09

	Outcome: probability of teaching in a high-achieving school (t+2, teachers in disadvantaged schools at baseline, winning middle award)	0.10	0.11
	Outcome: probability of teaching in a high-achieving school (t+1, teachers in more advantaged schools at baseline, winning lowest award)	0.44***	0.16
	Outcome: probability of teaching in a high-achieving school (t+2, teachers in more advantaged schools at baseline, winning lowest award)	0.38**	0.17
	Outcome: probability of teaching in a high-achieving school (t+1, teachers in more advantaged schools at baseline,	0.36***	0.11

	winning middle award)		
	Outcome: probability of teaching in a high-achieving school (t+2, teachers in more advantaged schools at baseline, winning middle award)	0.18	0.13
	Outcome: probability of teaching in a disadvantaged school (t+1, all teachers, winning lowest award)	-0.05	0.07
	Outcome: probability of teaching in a disadvantaged school (t+2, all teachers, winning lowest award)	0.05	0.07
	Outcome: probability of teaching in a disadvantaged school (t+1, all teachers, winning middle award)	-0.14***	0.07
	Outcome: probability of teaching in a	0.10	0.07

	disadvantaged school (t+2, all teachers, winning middle award)		
	Outcome: probability of teaching in a disadvantaged school (t+1, teachers in disadvantaged schools at baseline, winning lowest award)	-0.05	0.1
	Outcome: probability of teaching in a disadvantaged school (t+2, teachers in disadvantaged schools at baseline, winning lowest award)	0.21**	0.1
	Outcome: probability of teaching in a disadvantaged school (t+1, teachers in disadvantaged schools at baseline, winning middle award)	-0.02	0.07
	Outcome: probability of teaching in a disadvantaged	0.17*	0.09

	school (t+2, teachers in disadvantaged schools at baseline, winning middle award)		
	Outcome: probability of teaching in a disadvantaged school (t+1, teachers in more advantaged schools at baseline, winning lowest award)	-0.05	0.09
	Outcome: probability of teaching in a disadvantaged school (t+2, teachers in more advantaged schools at baseline, winning lowest award)	-0.09	0.11
	Outcome: probability of teaching in a disadvantaged school (t+1, teachers in more advantaged schools at baseline, winning middle award)	-0.22**	0.09
	Outcome: probability of teaching in a	0.05	0.11

							disadvantaged school (t+2, teachers in more advantaged schools at baseline, winning middle award)		
Hinze-Pifer and Méndez (2016)	Chile	Difficult Conditions Bonus for disadvantaged schools. Majority of beneficiary teachers receive between 4% to 10% over their base salary, with some receiving 15% or higher.	Regression discontinuity along the Difficult Conditions score - an index of measures of disadvantages including share of low-income students, distance from a large city, and public transit access.	Established in 1996.<br><br>Data is from 2008-2014. Schools apply to receive the bonus every two years.	2,032 schools (varies over time)	No significant impact on teacher retention; reduced working hours of teachers receiving the bonus	Outcome: end of year retention (%)	-0.438	0.371
							Outcome: contract hours	-0.264 ***	0.092
							Outcome: experience (years)	-0.066	0.187
							Outcome: teacher hours per student	-0.123 *	0.068
							Outcome: contract hours (t + 1)	-0.164	0.104
							Outcome: teacher hours per student (t + 1)	-0.168 **	0.075
Pugatch and Schroeder (2014)	Gambia	Hardship allowance that provides 30-40% bonus to teachers in remote locations.	Difference-in-differences and regression discontinuity along the 3-km threshold distance from a main road.	Allowance policy was adopted in 2005.<br><br>Data is from 2001 and 2003 (pre-treatment years) and 2010-2012 (post-treatment).	244 schools, of which148 are hardship schools and 96 are non-hardship schools	Increased share of qualified/certified teachers and pupil/qualified teacher ratio (RD analysis). The intervention did not increase share of female teachers.	Outcome: share of qualified teachers (%) (DD)	0.10 ***	0.03
							Outcome: share of female teachers (%) (DD)	-0.01	0.02
							Outcome: pupil-qualified teacher ratio (DD)	-0.02	9.3
							Outcome: share of qualified teachers (%) (RD)	0.16	0.11

							Outcome: share of female teachers (%) (RD)	0.08	0.09
							Outcome: pupil-qualified teacher ratio (RD)	-27.4 **	12.7
Rosa (2019)	Brazil	San Paolo city pays "wage-premiums [5%-7%] for teachers working in schools in selected neighborhoods" farther from downtown.<br><br>Area 1: no wage premium<br>Area 2: wage-premium of about 5%<br>Area 3: wage-premium of 7%	Regression discontinuity along the distance threshold (school to neighborhood boundaries).	Classification of neighborhoods and corresponding wage premium was set up in 1991.<br><br>Evaluation data from 2010.	546 schools	Wage premium has no effect on teachers' choice of school, still preferring schools with better environment and student outcomes.	Outcome: probability of teachers choosing to be in wage-premium schools (Area 2 vs Area 1) (using school latitude and longitude)	-0.0004	0.0011
							Outcome: probability of teachers choosing to be in wage-premium schools (Area 2 vs Area 1) (using school distance to boundary)	-0.0001	0.0011
							Outcome: probability of teachers choosing to be in wage-premium schools (Area 3 vs Area 2) (using school latitude and longitude)	0.0004	0.0011
							Outcome: probability of teachers choosing to be in wage-premium schools (Area 3 vs	0.0004	0.0010

							Area 2) (using school distance to boundary)		
Swai (2013)	Tanzania	Rukwa Civil Servant Facilitation Fund in Tanzania provides incentive to attract secondary school teachers to the region (cash incentive including signing bonus equivalent to at least one month take home pay, accommodations, and other inputs).	Matching. Retention rates in Rukwa which implements the rural incentive is compared against Kigoma, a neighboring region with no incentive policy in place.	Policy initially implemented in 2004. Data collection/ interviews were held in 2012.	290 teachers from Rukwa and 266 teachers from Kigoma	Retention in rural Rukwa is higher than in rural Kigoma but not statistically different. Within Rukwa, no significant difference in retention rate between teachers recruited through the incentive system and teachers recruited via traditional means.	Outcome: % of teachers who stayed more than 3 years (Rukwa vs Kigoma)	0.002	No statistical significance reported.
							Outcome: % of teachers employed in Rukwa who stayed more than 3 years (recruited via the incentive system vs recruited via traditional route)	0.003	Not statistically significant.
Urquiola and Vegas (2005)	Bolivia	Salary bonus of about 12.5 percent of total compensation available. Additional bonus is available for teachers depending on seniority and trainings received.	Matching between rural teachers and urban teachers in three cities (La Paz/El Alto, Cochabamba, and Santa Cruz)	Multiple geography bonus with no clear timelines, but the study reflects 2002 salary rates.	1,606 schools from the three cities	Number of hours worked and probability of holding a second job decreased.	Outcome: total hours worked (all cities, no control)	-.7.2 ***	0.5
							Outcome: total hours worked (all cities, controlling for teachers and school characteristics)	-.7.3 ***	0.5
							Outcome: total hours worked (all cities, controlling for teachers and school characteristics, with dummy for teacher experience)	-4.2 ***	0.4

	Outcome: probability of holding more than one job (all cities, no control)	-0.16 ***	0.01
	Outcome: probability of holding more than one job (all cities, with dummy for teacher training)	-0.16 ***	0.01
	Outcome: probability of holding more than one job (all cities, with dummy for teacjer training, with dummy for teacher experience)	-0.09 ***	0.01

Note: * significant at 10%; ** significant at 5%; *** significant at 1%.

Table A.6

Vote Count of Teacher-Related Outcomes from the 15 Eligible Studies

	Positive, significant	Positive, insignificant	Negative, insignificant	Negative, significant	Total
All teacher outcomes	44	33	35	16	128
	34%	26%	27%	13%	100%
Teacher preferences	12	6	6	0	24
	50%	25%	25%	0%	100%
Teacher placement	14	10	16	10	50
	28%	20%	32%	20%	100%
Teacher turn-over	11	12	8	0	31
	35%	39%	26%	0%	100%
Teacher competence	4	5	4	0	13
	31%	38%	31%	0%	100%
Teacher working hours/ probability of holding a second job	3	0	1	6	10
	30%	0%	10%	60%	100%

Table A.7

Association of the size of incentive and how rural a country is to the impact of the incentives evaluated in those countries.

VARIABLES	(1) Incentive has positive impact	(2) Incentive has positive and significant impact	(3) Incentive has positive impact	(4) Incentive has positive and significant impact	(5) Incentive has positive impact	(6) Incentive has positive and significant impact
Size of incentive	0.024	0.041***				
	(0.014)	(0.011)				
Share of population living 1 hour outside of cities			0.020	-0.012		
			(0.018)	(0.022)		
Share of population living 2 hours outside of cities					0.039	-0.017
					(0.063)	(0.074)
Constant	0.287	-0.155	0.495	0.892**	0.673**	0.755**
	(0.308)	(0.261)	(0.308)	(0.371)	(0.248)	(0.289)
Observations	10	10	10	10	10	10
R-squared	0.289	0.612	0.131	0.039	0.045	0.006

Note: We use the data from Cattaneo et al. (2021) to calculate the share of population living outside of cities in the countries where the studies are conducted. Of the 12 financial incentive studies, we exclude two studies that have mixed impact. Seven of the remaining studies have positive and significant impact on teacher recruitment and retention while one study has a positive but not statistically significant impact (Swai et al. 2013).
Standard errors in parentheses. *** p<0.01, ** p<0.05, * p<0.1

Table A.8

Student Outcomes from Studies on Incentivizing Teachers in Hard-to-Staff Schools

Study	Country	Intervention	Research Design	Timeframe	Data/Sample	Outcomes	Specification	Coef.	SE/CI
Bobba et al. (2022)	Peru	Government-sponsored wage bonus for teachers in select rural areas categorized by population and travel time to provincial capital computed by GPS. Bonus is up to S/500, equivalent to up to 30 percent of contract teachers' monthly earnings.	Regression discontinuity using population and distance cut-off.	Policy was first implemented in 2014 with increase in bonuses in 2015.<br><br>Study period is 2015-2018	The main results table report outcomes based on data from up to 925 schools.	Students in program schools performed better, especially students in schools with short-term vacancies, a potential result of the improved competence of newly recruited contractual teachers (see teacher outcomes in Appendix A1).	Outcome: Spanish test (z-score) (any vacancy)	0.298 **	0.127
							Outcome: Spanish test (z-score) (permanent vacancy)	-0.057	0.190
							Outcome: Spanish test (z-score) (short-term vacancy)	0.317 **	0.137
							Outcome: Math test (z-score) (any vacancy)	0.350 **	0.142
							Outcome: Math test (z-score) (permanent vacancy)	-0.047	0.248
							Outcome: Math test (z-score) (short-term vacancy)	0.470 ***	0.159
Cabrera and Webbink (2020)	Uruguay	Contexto Socio Cultural Crític (CSCC program) provides up to 26% increase in base salary for teachers working	Regression discontinuity around the poverty index threshold.	The program was launched in 1995 and updated in 2005 to use a poverty index cut-off as	543 schools	Small effect on student performance (test score, attendance, grade-	Outcome: insufficient student attendance (+/- 1.5 pts discontinuity)	-0.052	0.046
							Outcome: student grade retention (+/- 1.5 pts discontinuity)	0.014	0.038

		poor neighborhoods		eligibility criteria.<br><br>Outcome variables are available until 2013 with administrative data available since 1992 (study does not evaluate long-term effects, but outcomes against program participation in year t-1 and t-2)		retention, dropout)	Outcome: student dropout (+/- 1.5 pts discontinuity)	0.039	0.061
							Outcome: student math test scores	-0.131	0.089
							Outcome: insufficient student attendance (+/- 1.0 pts discontinuity)	-0.153 ***	0.055
							Outcome: student grade retention (+/- 1.0 pts discontinuity)	-0.007	0.047
							Outcome: student dropout (+/- 1.0 pts discontinuity)	-0.056	0.066
Camelo and Ponczek (2021)	Brazil	ALE program (Adicional por Local de Exercício): "wage premium (24% to 36%) to teachers at disadvantaged schools" based on school location	Regression discontinuity around the socio-economic index threshold.	Program was launched in 2008.<br><br>Data for analysis covers 2007 to 2012.	1,422 schools received compensation and 1,324 did not	No effect on average test score but the program reduced proportion of low-performers as measured by test scores.	Outcome: student proficiency scores in Math (2008)	-0.090	0.085
							Outcome: student proficiency scores in Math (2009)	0.110	0.080
							Outcome: student proficiency scores in Math (2010)	-0.009	0.087
							Outcome: student proficiency scores in Math (2011)	0.046	0.104
							Outcome: student proficiency scores in Math (2012)	0.029	0.077

Outcome: student proficiency scores in Math (pooled)	0.024	0.071
Outcome: student proficiency scores in Reading (2008)	-0.063	0.073
Outcome: student proficiency scores in Reading (2009)	0.053	0.073
Outcome: student proficiency scores in Reading (2010)	-0.034	0.075
Outcome: student proficiency scores in Reading (2011)	0.026	0.107
Outcome: student proficiency scores in Reading (2012)	0.000	0.068
Outcome: student proficiency scores in Reading (pooled)	-0.002	0.064
Outcome: % of low performers in Math (2008)	-0.023	0.033
Outcome: % of low performers in Math (2009)	-0.081 **	0.039
Outcome: % of low performers in Math (2010)	-0.068 *	0.039
Outcome: % of low performers in Math (2011)	-0.085 **	0.042

							Outcome: % of low performers in Math (2012)	-0.083 **	0.040
							Outcome: % of low performers in Math (pooled)	-0.068 **	0.033
							Outcome: % of low performers in Reading (2008)	-0.036	0.026
							Outcome: % of low performers in Reading (2009)	-0.050 **	0.025
							Outcome: % of low performers in Reading (2010)	-0.051	0.032
							Outcome: % of low performers in Reading (2011)	-0.064 *	0.037
							Outcome: % of low performers in Reading (2012)	-0.064 **	0.031
							Outcome: % of low performers in Reading (pooled)	-0.054 **	0.025
Castro and Esposito (2022)	Peru	Rural bonuses based on community population and distance from capital	Regression discontinuity along the school's distance in minutes to the nearest provincial capital (120 minutes) and	Started in 1990, revamped in 2014 to increase bonuses and include more schools.	9,948 extremely rural schools (treatment group; receives the highest available bonus)<br><br>11,575	No clear evidence of impact on test scores.	Outcome: math test scores (standard deviation) (schools in communities with less than 500 inhabitants)	-0.056	0.066
							Outcome: math test scores in control schools less than 30 minutes away	0.037	0.071

	number of inhabitants (500)	Evaluated in 2016.	rural schools (control; receives smaller bonus)		(standard deviation) (schools in communities with less than 500 inhabitants)		
					Outcome: math test scores (standard deviation) (schools in communities with less than 500 inhabitants)	-0.239 *	0.123
					Outcome: math test scores in control schools less than 30 minutes away (standard deviation) (schools in communities with less than 500 inhabitants)	0.277 **	0.126
					Outcome: reading comprehension test scores (standard deviation) (schools in communities more than 120 minutes away from provincial capital)	-0.096	0.071
					Outcome: reading comprehension test scores in control schools less than 30 minutes away (standard deviation) (schools in	0.031	0.069

							communities more than 120 minutes away from provincial capital)		
							Outcome: reading comprehension test scores (standard deviation) (schools in communities more than 120 minutes away from provincial capital)	-0.169	0.110
							Outcome: reading comprehension test scores in control schools less than 30 minutes away (standard deviation) (schools in communities more than 120 minutes away from provincial capital)	0.299 ***	0.112
Chelwa et al. (2019)	Zambia	Rural hardship allowance corresponding to a salary increase of 20% "allocated to schools outside a given radius from district centers."	Regression discontinuity by GPS-computed distance to district city centers.	Allowance was implemented in the 1990s, revamped in 2008 (to become a 20% salary increase) and some implementation rules were changed in 2010.	3,000 schools, about half received the allowance allocation; final sample is 137 schools (44 pairs across the threshold) due to challenges in implementation	Some evidence of positive impact (significant for boys).	Outcome: proportion of students who score a Division One category - highest level category (boys only)	0.02 *	0.011
							Outcome: proportion of students who score a Division One category - highest level category (girls only)	0.009	0.011

				Evaluation uses data from 2014 to 2015	and data availability				
Chin (2005)	India	Operation Blackboard is a government initiative to recruit additional teachers for one-teacher primary schools, complemented with provision of equipment packets (blackboards, books, maps, charts, toys, teacher's manuals, and other basic inputs) to all primary schools.	Difference-in-differences by birth cohort (children who started attending primary schools before and after 1987 when the program was launched) and state of residence (children in states with higher share of one-teacher schools were more exposed to the program)	Launched in 1987, the program served all originally targeted schools by 1994.	Recruitment drive for 140,000 teachers as second teachers to one-teacher primary schools	Positive impact on primary completion rate especially for girls and poor students.	Outcome: primary school completion rate (girls, basic controls)	0.0161 ***	0.0029
							Outcome: primary school completion rate (girls, household controls)	0.0165 ***	0.0026
							Outcome: primary school completion rate (girls, region-specific trend)	0.0120 ***	0.0028
							Outcome: primary school completion rate (girls, state-specific trend)	0.0093 **	0.0044
							Outcome: primary school completion rate (girls, state program x year of birth)	0.0091 **	0.0044
							Outcome: primary school completion rate (boys, basic controls)	0.0164 ***	0.0028
							Outcome: primary school completion rate (boys, household controls)	0.0144 ***	0.0026

Outcome: primary school completion rate (boys, region-specific trend)	0.0100 ***	0.0027
Outcome: primary school completion rate (boys, state-specific trend)	0.0034	0.0045
Outcome: primary school completion rate (boys, state program x year of birth)	0.0027	0.0045
Outcome: primary school completion rate (girls, household controls, bottom quartile of household expenditure)	0.0295 ***	0.0040
Outcome: primary school completion rate (girls, region-specific trend, bottom quartile of household expenditure)	0.0248 ***	0.0041
Outcome: primary school completion rate (girls, state-specific trend, bottom quartile of household expenditure)	0.0216 ***	0.0054
Outcome: primary school completion rate (girls, state program x year of	0.0223 ***	0.0054

							birth, bottom quartile of household expenditure)		
							Outcome: primary school completion rate (boys, household controls, bottom quartile of household expenditure)	0.0196 ***	0.0040
							Outcome: primary school completion rate (boys, region-specific trend, bottom quartile of household expenditure)	0.0149 ***	0.0042
							Outcome: primary school completion rate (boys, state-specific trend, bottom quartile of household expenditure)	0.0070	0.0056
							Outcome: primary school completion rate (boys, state program x year of birth, bottom quartile of household expenditure)	0.0078	0.0055
Hinze-Pifer and Méndez (2016)	Chile	Difficult Conditions Bonus for "disadvantaged" schools. Majority of	Regression discontinuity along the Difficult Conditions score - an	Established in 1996.<br><br>Data is from 2008-2014. Schools apply	2032 schools (varies over time)	No significant impact on performance outcomes	Outcome: 4th Grade math score	-0.0194	0.0125
							Outcome: 4th Grade reading score	-0.0132	0.0119
							Outcome: 4th Grade math score (t + 1)	-0.0212	0.0135

		beneficiary teachers receive between 4% to 10% over their base salary, with some receiving 15% or higher.	index of measures of disadvantages including share of low-income students, distance from a large city, and public transit access.	to receive the bonus every two years.			Outcome: 4th Grade reading score (t+1)	- 0.00948	0.0124
Pugatch and Schroeder (2018)	Gambia	Hardship allowance that provides 30-40% bonus to school teachers in remote locations.	Difference-in-differences and regression discontinuity along the 3-km threshold distance from a main road to be classified as hardship area.	Allowance policy was adopted in 2005.<br><br>Data for evaluation is from 2012	244 schools, of which148 are hardship schools and 96 are non-hardship schools	No significant impact on performance outcomes except for baseline high performers	Outcome: 4th Grade math score	-0.0194	0.0125
							Outcome: 4th Grade reading score	-0.0132	0.0119
							Outcome: 4th Grade math score (t + 1)	-0.0212	0.0135
							Outcome: 4th Grade reading score (t+1)	- 0.00948	0.0124
							Outcome: Grade 3 (all, English)	0.07	0.09
							Outcome: Grade 3 (boys, English)	0.07	0.12
							Outcome: Grade 3 (girls, English)	0.08	0.09
							Outcome: Grade 5 (all, English)	0.23	0.17
							Outcome: Grade 5 (boys, English)	0.21	0.22
							Outcome: Grade 5 (girls, English)	0.25 *	0.14
							Outcome: Grade 3 (all, Math)	0.02	0.12

Outcome: Grade 3 (boys, Math)	0.01	0.13
Outcome: Grade 3 (girls, Math)	0.02	0.12
Outcome: Grade 5 (all, Math)	0.20	0.16
Outcome: Grade 5 (boys, Math)	0.16	0.20
Outcome: Grade 5 (girls, Math)	0.22	0.15
Outcome: at or below median of the socio-economic status index which includes family support in education and parents' educational status (all, English)	0.09	0.17
Outcome: at or below median of the socio-economic status index (boys, English)	-0.04	0.23
Outcome: at or below median of the socio-economic status index (girls, English)	0.18	0.13
Outcome: above median of the socio-economic status index (all, English)	0.38 *	0.20
Outcome: above median of the socio-economic status index (boys, English)	0.45 *	0.26

	Outcome: above median of the socio-economic status index (girls, English)	0.33 *	0.18
	Outcome: at or below median of the socio-economic status index which includes family support in education and parents’ educational status (all, Math)	0.01	0.16
	Outcome: at or below median of the socio-economic status index (boys, Math)	-0.09	0.20
	Outcome: at or below median of the socio-economic status index (girls, Math)	0.10	0.16
	Outcome: above median of the socio-economic status index (all, Math)	0.41 **	0.19
	Outcome: above median of the socio-economic status index (boys, Math)	0.44 *	0.24
	Outcome: above median of the socio-economic status index (girls, Math)	0.40 **	0.17

Urquiola and Vegas (2005)	Bolivia	Salary bonus of about 12.5 percent of total compensation available. Additional bonus is available for teachers depending on seniority and trainings received.	Matching between rural teachers and urban teachers in three cities (La Paz/El Alto, Cochabamba, and Santa Cruz)	Multiple geography bonus with no clear timelines, but the study reflects 2002 salary rates.	1,606 schools from the three cities	No significant impact on test scores and grade repetition; slightly positive and inconsistently significant effect on pass rate and dropout rate	Outcome: third-grade language score (no control)	-0.10	0.12
							Outcome: third-grade language score (controlling for teacher and school characteristics)	-0.11	0.12
							Outcome: third-grade math score (no control)	0.06	0.12
							Outcome: third-grade math score (controlling for teacher and school characteristics)	0.09	0.12
							Outcome: repetition rate (no control)	-0.006 **	0.003
							Outcome: repetition rate (controlling for teacher and school characteristics)	-0.004	0.003
							Outcome: pass rate (no control)	0.014 ***	0.004
							Outcome: pass rate (controlling for teacher and school characteristics)	[+] ***	0.004
							Outcome: dropout rate (no control)	0.009 ***	0.001
							Outcome: dropout rate (controlling for teacher and school characteristics)	0.006 ***	0.001

Note: * significant at 10%; ** significant at 5%; *** significant at 1%. [+] This coefficient is reported as 0.00 and yet is marked at highly statistically significant. In a subset of the sample, adding controls reduces the coefficient by 0.001, so this estimate may be close to 0.013 (i.e., one less than the coefficient above it).

Table A.9

Vote Count of Student-Related Outcomes from the 15 Eligible Studies

	Positive, significant	Positive, insignificant	Negative, insignificant	Negative, significant	Total
All student outcomes	43	36	23	1	103
	42%	35%	22%	1%	100%
Student achievement	26	31	21	1	79
	33%	39%	27%	1%	100%
Student attendance	17	5	2	0	24
	71%	21%	8%	0%	100%

Figure A.1

Proportion of Students with At Least One Exercise Book in Urban and Semi-Urban vs. Rural Areas in Selected Low- and Middle-Income Countries

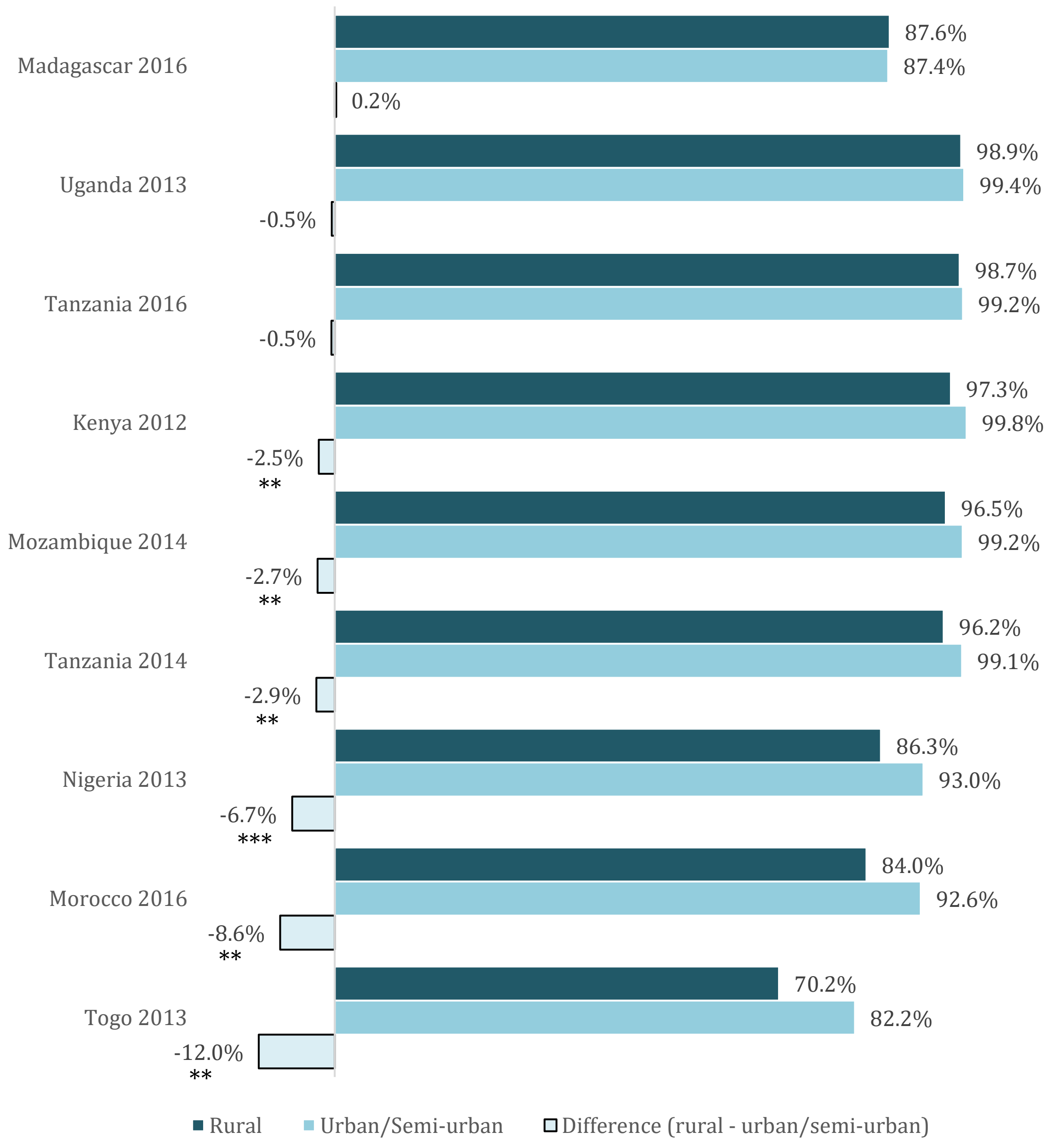

Source: Authors' analysis using data from the World Bank's Service Delivery Indicators Education Survey.
Note: Averages are first computed at the school-level and aggregated at the country level using school-level weights. Differences in rural and urban/semi-urban rates are tested using bivariate regressions with school-level weights and robust standard errors and are presented here with statistical significance: * significant at 10%; ** significant at 5%; *** significant at 1%.

Figure A.2

Proportion of Classrooms with Functioning Boards in Urban and Semi-Urban vs. Rural Areas in Selected Low- and Middle-Income Countries

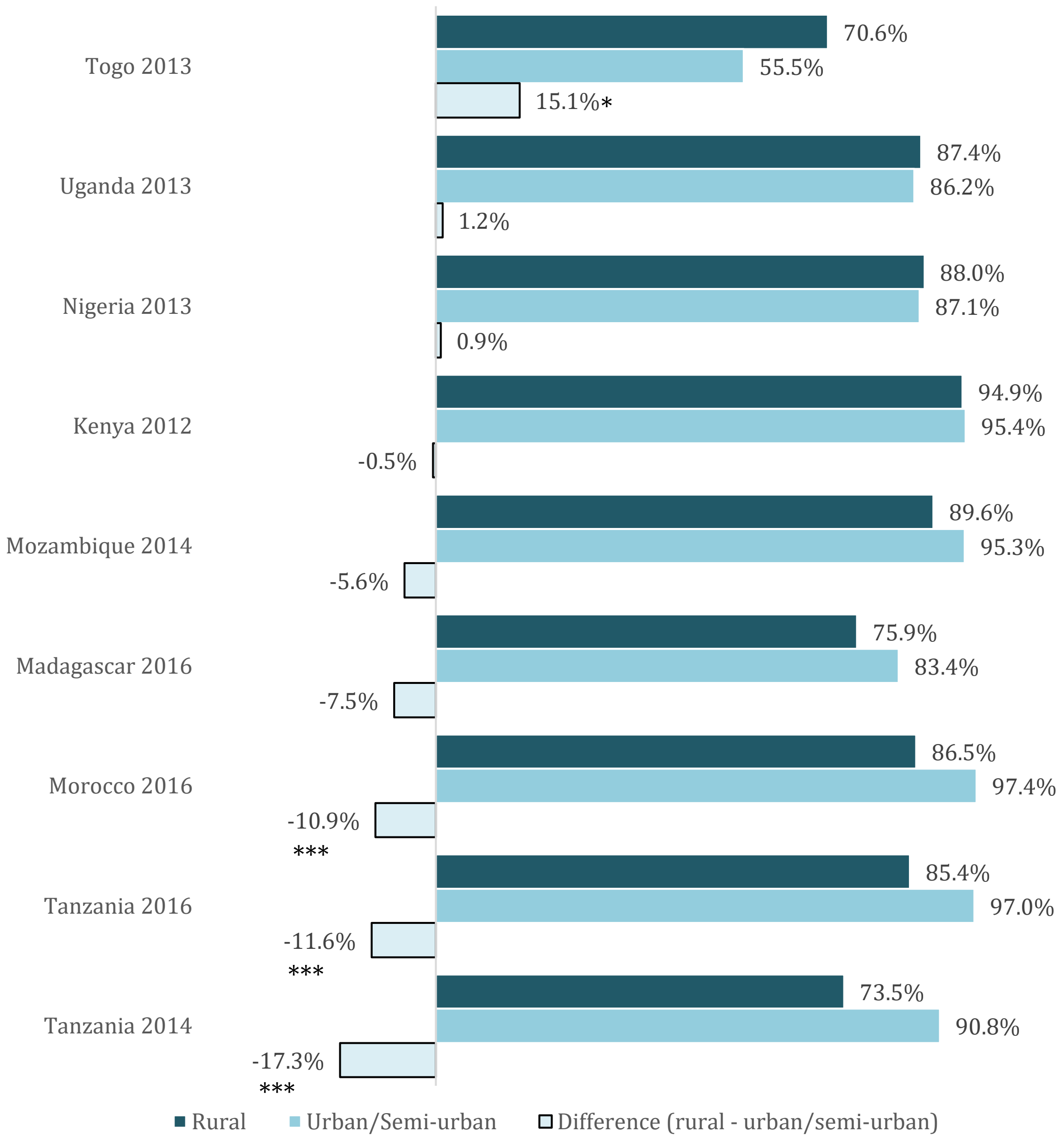

Source: Authors' analysis using data from the World Bank's Service Delivery Indicators Education Survey.
Note: Averages are first computed at the school-level and aggregated at the country level using school-level weights. Differences in rural and urban/semi-urban rates are tested using bivariate regressions with school-level weights and robust standard errors and are presented here with statistical significance: * significant at 10%; ** significant at 5%; *** significant at 1%.

Figure A.3

Impact on teacher recruitment and retention against size of financial incentive.

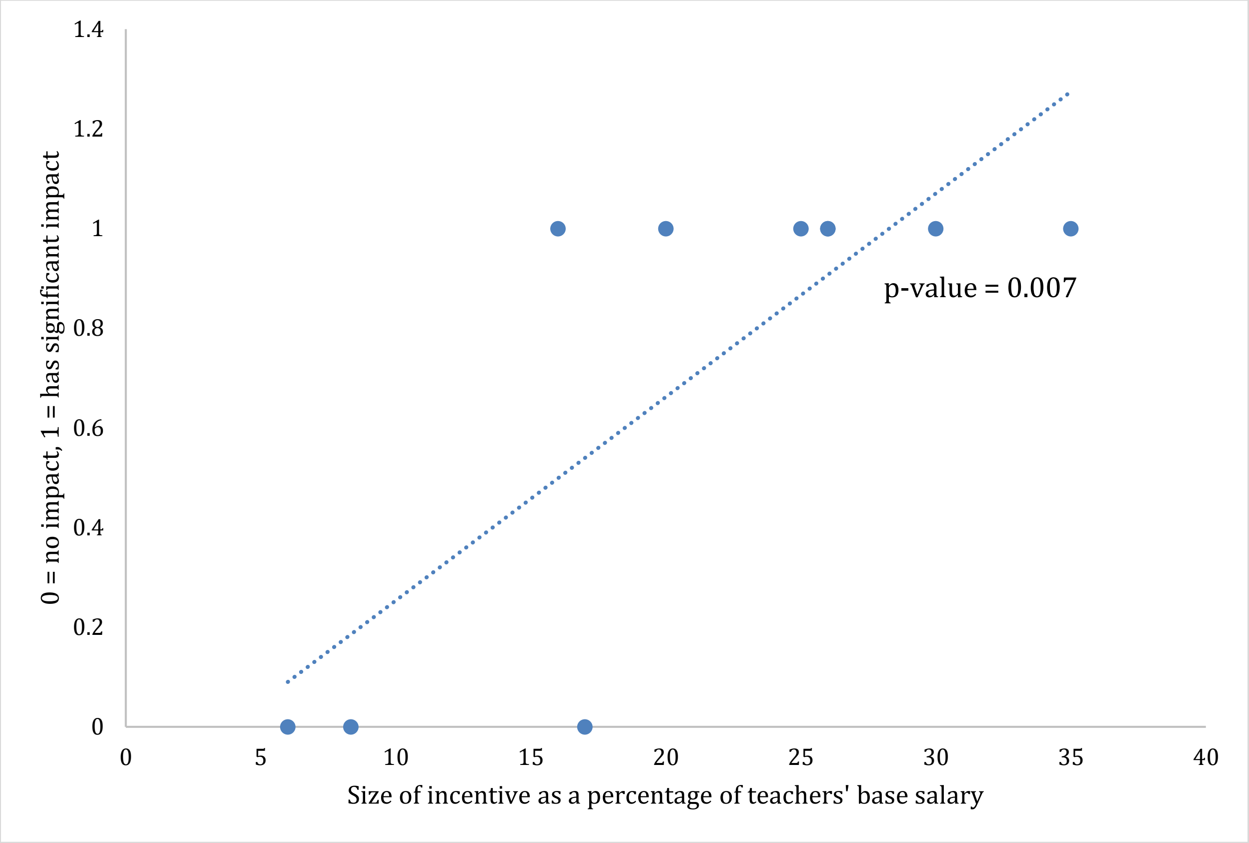

Note: We plotted the size of financial incentives as a percentage of teachers' base salary (see Table 5) against an indicator of whether the incentive has positive and significant impact on teacher recruitment or retention and ran a simple bivariate regression on the two variables. We used the midpoint value for financial incentives that are given as a range (i.e. for the incentive described as 24 percent to 36 percent of base salary, we use the midpoint value — 30 percent). The regression and the scatterplot above shows a positive association between the size of incentive and the likelihood the incentive has a positive and significant impact on teacher recruitment and retention.

Figure A.4

Share of schools in rural, urban and semi-urban locations.

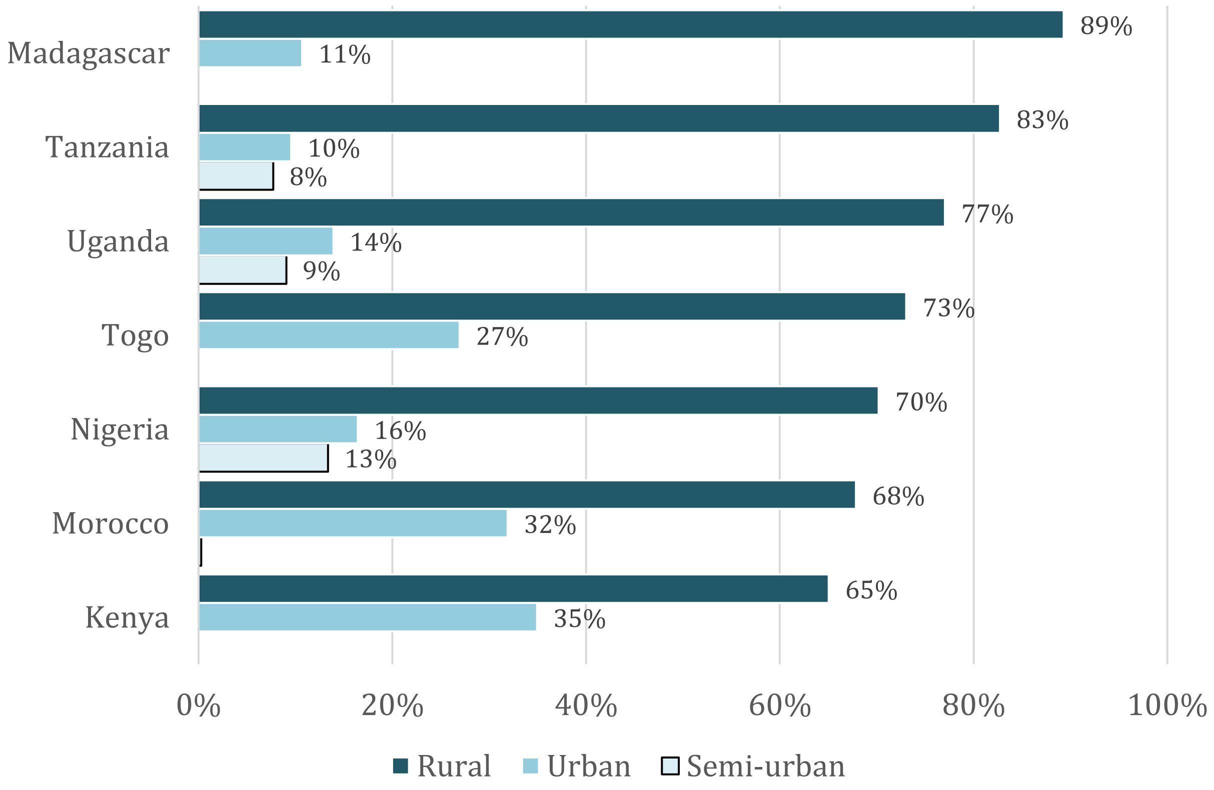

Source: Authors' analysis using data from the World Bank's Service Delivery Indicators education surveys.

Figure A.5

Share of population in each country according to distance to urban centers.

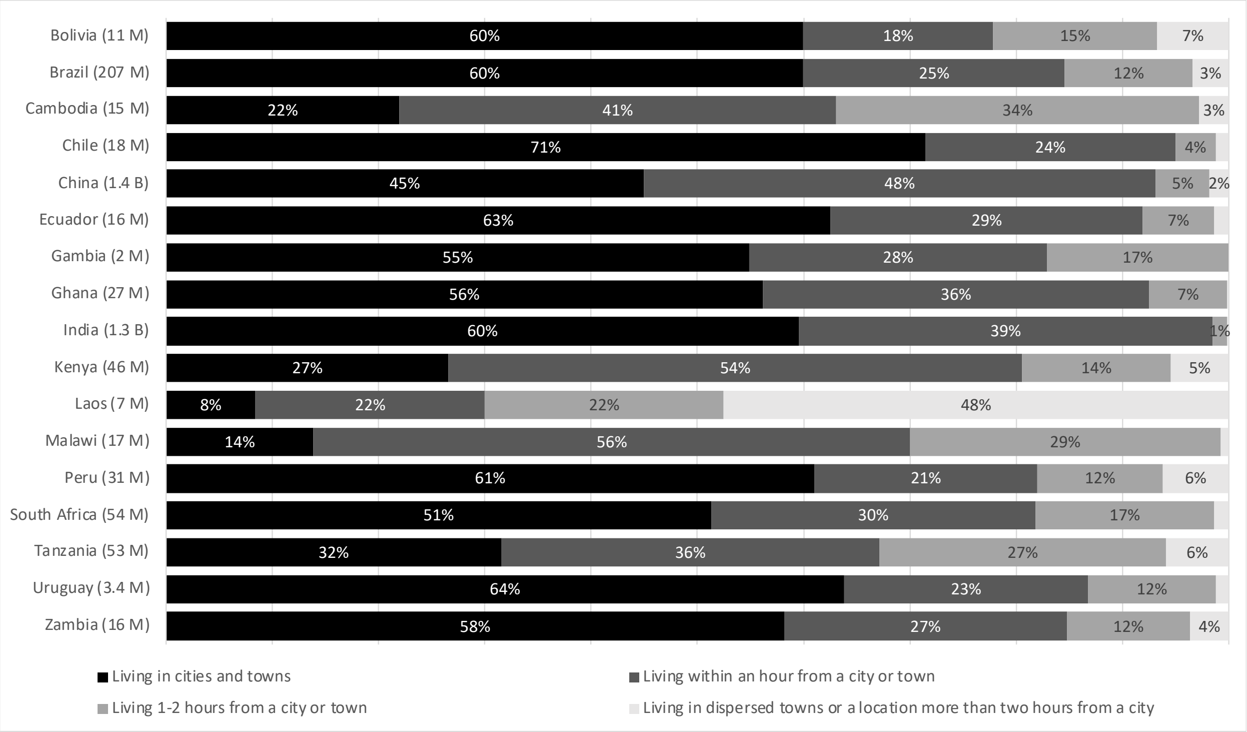

Source: Adapted from Cattaneo et al. (2021). Cities and towns are defined here as urban agglomerations with inhabitants greater than 5,000. Other cluster areas with inhabitants less than 5,000 are considered dispersed towns. To provide additional context, the total country populations in 2015—the same year as the underlying data in this chart—are inside parentheses next to country names.

Appendix Section A.6: Appendix References

Ajzenman, N., Bertoni, E., Elacqua, G., Marotta, L., Méndez Vargas, C., 2021a. Altruism or Money?: Reducing Teacher Sorting Using Behavioral Strategies in Peru (IZA DP No. 14111). IZA institute of Labor Economics.

Ajzenman, N., Elacqua, G., Marotta, L., Olsen, A.S., 2021b. Order effects and employment decisions: Experimental evidence from a nationwide program.

Alfonso, M., Santiago, A., Bassi, M., 2010. Estimating the Impact of Placing Top University Graduates in Vulnerable Schools in Chile (No. Technical Note IDB-TN-230). Inter-American Development Bank.

Banerjee, A.V., Cole, S., Duflo, E., Linden, L., 2007. Remedying Education: Evidence from Two Randomized Experiments in India. Q J Econ 122, 1235–1264. https://doi.org/10.1162/qjec.122.3.1235

Bobba, M., Ederer, T., Leon-Ciliotta, G., Neilson, C., Nieddu, M.G., 2022. Teacher Compensation and Structural Inequality: Evidence from Centralized Teacher School Choice in Peru (Working Paper No. 29068).

Bold, T., Gauthier, B., Svensson, J., Wane, W., 2010. Delivering service indicators in education and health in Africa : a proposal (No. WPS5327). World Bank.

Bold, T., Kimenyi, M., Mwabu, G., Ng'ang'a, A., Sandefur, J., 2018. Experimental evidence on scaling up education reforms in Kenya. Journal of Public Economics 168, 1–20. https://doi.org/10.1016/j.jpubeco.2018.08.007

Bold, T., Svensson, J., Gauthier, B., Mæstad, O., Wane, W., 2011. Service delivery indicators: Pilot in education and health care in Africa (No. CMI Report R 2011:8). Chr. Michelsen Institute, Bergen, Norway.

Cabrera, J.M., Webbink, D., 2020. Do Higher Salaries Yield Better Teachers and Better Student Outcomes? J. Human Resources 55, 1222–1257. https://doi.org/10.3368/jhr.55.4.0717-8911R3

Camelo, R., Ponczek, V., 2021. Teacher Turnover and Financial Incentives in Underprivileged Schools: Evidence from a Compensation Policy in a Developing Country. Economics of Education Review 80, 102067. https://doi.org/10.1016/j.econedurev.2020.102067

Castro, J.F., Esposito, B., 2022. The Effect of Bonuses on Teacher Retention and Student Learning in Rural Schools: A Story of Spillovers. Education Finance and Policy 1–59. https://doi.org/10.1162/edfp_a_00348

Cattaneo, A., Nelson, A., McMenomy, T., 2021. Global mapping of urban–rural catchment areas reveals unequal access to services. Proceedings of the National Academy of Sciences 118, e2011990118. https://doi.org/10.1073/pnas.2011990118

Chacón, A., Peña, P., 2017. The impact of Enseña por México on student socioemotional skills. Microanalitica.

Chelwa, G., Pellicer, M., Maboshe, M., 2019. Teacher Pay and Educational Outcomes: Evidence from the Rural Hardship Allowance in Zambia. South African Journal of Economics 87, 255–282. https://doi.org/10.1111/saje.12227

Chin, A., 2005. Can redistributing teachers across schools raise educational attainment? Evidence from Operation Blackboard in India. Journal of Development Economics 78, 384–405. https://doi.org/10.1016/j.jdeveco.2004.09.004

Cobbold, C., 2006. Attracting and retaining rural teachers in Ghana: the premise and promise of a district sponsorship scheme. Journal of Education for Teaching 32, 453–469. https://doi.org/10.1080/02607470600982142

Cumsille, B.R., Fiszbein, A., 2015. Crème de la Crème: The Teach For All Experience and Its Lessons for Policy-Making in Latin America. education policy analysis archives 23, 46–46. https://doi.org/10.14507/epaa.v23.1810
Duflo, E., Dupas, P., Kremer, M., 2015. School governance, teacher incentives, and pupil–teacher ratios: Experimental evidence from Kenyan primary schools. Journal of Public Economics 123, 92–110. https://doi.org/10.1016/j.jpubeco.2014.11.008
Elacqua, G., Hincapie, D., Hincapie, I., Montalva, V., 2022. Can Financial Incentives Help Disadvantaged Schools to Attract and Retain High-Performing Teachers? Evidence from Chile. Journal of Policy Analysis and Management n/a. https://doi.org/10.1002/pam.22375
Fagernäs, S., Pelkonen, P., 2012. Preferences and Skills of Indian Public Sector Teachers. IZA Journal of Labor & Development 1, 1–31.
Gad, B.K., 2015. Recruitment and Retention of Public Sector Teachers in Ghana: A Discrete Choice Experiment. University of Ghana.
Hinze-Pifer, R., Méndez, C., 2016. Evaluating the Impact of Supplemental Funds on Disadvantaged Schools: Evidence from Chile. University of Chicago.
Kamere, I., Makatiani, M.I., Nzau, A.K., 2019. Policy interventions for attraction and retention of female teachers in rural secondary schools: Perspectives of rural educators in Makueni County, Kenya. MSINGI JOURNAL of the Department of Educational Foundations 1, 50–59.
Lavado, P., Guzmán, R., 2020. Evaluación de impacto de enseña Perú.
Li, J., Xue, E., 2021. "Teach to adapt or adapt to teach": qualitative study on the new "special-post teachers" in China's rural schools. Educational Philosophy and Theory 53, 1295–1305. https://doi.org/10.1080/00131857.2020.1840350
Liao, W., Liu, Y., Zhao, P., Li, Q., 2019. Understanding how local actors implement teacher rotation policy in a Chinese context: a sensemaking perspective. Teachers and Teaching 25, 855–873. https://doi.org/10.1080/13540602.2019.1689490
Mwenda, D.B., Mgomezulu, V.Y., 2018. Impact of Monetary Incentives on Teacher Retention in and Attraction to Rural Primary Schools: Case of the Rural Allowance in Salima District of Malawi. African Educational Research Journal 6, 120–129.
Poti, J., Mutsvangwa, A., Hove, M.L., 2014. Teacher Retention and Quality Education: Impact of Rural Incentives in North-West, South Africa. Mediterranean Journal of Social Sciences 5, 792.
Pugatch, T., Schroeder, E., 2018. Teacher pay and student performance: evidence from the Gambian hardship allowance. Journal of Development Effectiveness 10, 249–276. https://doi.org/10.1080/19439342.2018.1452778
Pugatch, T., Schroeder, E., 2014. Incentives for teacher relocation: Evidence from the Gambian hardship allowance. Economics of Education Review 41, 120–136. https://doi.org/10.1016/j.econedurev.2014.04.003
Rosa, L., 2019. Teacher Preferences in Developing Countries: Evidences from the City of Sao Paulo, Brazi.
See, B.H., Morris, R., Gorard, S., El Soufi, N., 2020. What works in attracting and retaining teachers in challenging schools and areas? Oxford Review of Education 46, 678–697. https://doi.org/10.1080/03054985.2020.1775566
Service Delivery Indicators, 2017. SDI Definition of Indicators - Education [WWW Document]. URL https://www.sdindicators.org/indicators

Sisouphanthong, V., Suruga, T., Kyophilavong, P., 2020. Valuation of incentives to recruit and retain teachers in rural schools: Evidence from a choice experiment in Cambodia and Laos. Cogent Education 7, 1724243. https://doi.org/10.1080/2331186X.2020.1724243

Swai, A., 2013. The Effects of Incentive Initiatives on Teacher Retention in Tanzania: A Case of the Rukwa Region. Master's Capstone Projects.

Teach for All, 2021. Teach for All Network Partners [WWW Document]. URL https://teachforall.org/network-partners

UNESCO, 2020. A review of the use of contract teachers in sub-Saharan Africa. UNESCO.

Urquiola, M., Vegas, E., 2005. Arbitrary Variation in Teacher Salaries: An Analysis of Teacher Pay in Bolivia, in: Vegas, E. (Ed.), Incentives to Improve Teaching: Lessons from Latin America. The World Bank.

World Bank Microdata Library, 2021. Service Delivery Indicators [WWW Document]. URL https://microdata.worldbank.org/index.php/catalog/sdi

Yin, M.Y., Mu, G.M., 2020. Recognising localised pedagogical capital: a reflexive revisit of an alternative teacher preparation programme in China. Educational Philosophy and Theory 52, 1290–1301. https://doi.org/10.1080/00131857.2020.1738921

Yin, Y.M., Dooley, K., Mu, G.M., 2019. Why do graduates from prestigious universities choose to teach in disadvantaged schools? Lessons from an alternative teacher preparation program in China. Teaching and Teacher Education 77, 378–387. https://doi.org/10.1016/j.tate.2018.10.011

Zhai, X., 2019. Becoming a teacher in rural areas: How curriculum influences government-contracted pre-service physics teachers' motivation. International Journal of Educational Research 94, 77–89. https://doi.org/10.1016/j.ijer.2018.11.012
